# Pharmacokinetics, safety, and tolerability of fosmanogepix IV to oral switch and multiple IV doses in healthy participants

**DOI:** 10.1128/aac.01455-23

**Published:** 2024-03-29

**Authors:** Michael R. Hodges, Susan Hazel, William G. Kramer, Ewoud-Jan van Hoogdalem, Sjoerd van Marle, Margaret Tawadrous, Abhijeet Jakate

**Affiliations:** 1 Amplyx Pharmaceuticals, Inc., San Diego, California, USA; 2 Pfizer Inc., New York, New York, USA; 3 Kramer Consulting LLC, North Potomac, Maryland, USA; 4 ICON, Groningen, the Netherlands; University Children's Hospital Münster, Münster, Germany

**Keywords:** fosmanogepix (FMGX), manogepix (MGX), intravenous, invasive fungal diseases, antifungals

## Abstract

Fosmanogepix [FMGX, APX001; active form: manogepix (MGX), APX001A] is a first-in-class, intravenous (IV)/oral antifungal currently being evaluated for invasive fungal disease treatment. Data from two phase 1, placebo-controlled studies [IV–oral switch (study 1) and multiple IV doses (study 2)] evaluating FMGX tolerability, and pharmacokinetics (PK) are presented. Healthy adults (study 1: 18–65 years; study 2: 18–55 years) were eligible (randomized 3:1 to FMGX: placebo). Eleven participants completed study 1. In study 2, 51 participants (48 planned + 3 replacement) were enrolled in six cohorts (8 participants each; 34 completed the study). In study 1, overall MGX systemic exposures were comparable from day 1 to day 42 of dosing; steady-state plasma concentrations were achieved in ≤24 h following two IV loading doses (1,000 mg) and exposures maintained after switching [IV (600 mg) to daily oral doses (800 mg)]. FMGX was safe and well-tolerated. In study 2, FMGX IV doses (loading doses twice daily/maintenance doses once daily; 3-h infusion) of 1,500/900 mg (cohort A), 900/900 mg (cohort B), and 1,000/900 mg (cohort C: with ondansetron) were not well-tolerated; most participants reported nausea and infrequent vomiting. FMGX IV doses of 1,000/750 mg (cohort D), 1,000/850 mg (cohort E), and 1,000/900 mg (cohort F: ondansetron prn) were relatively better tolerated. Steady-state systemic exposures were achieved between days 2 and 4. All cohorts had similar geometric mean (GM) concentrations during maintenance dosing and similar GM PK parameters. Dosing regimen evaluated in study 1 was safe, well-tolerated, and may be used for future clinical evaluations.

## INTRODUCTION

Invasive fungal diseases (IFDs) are associated with 1.5 million deaths every year, the majority (>90%) of which are caused by *Candida*, *Aspergillus*, or *Cryptococcus* spp. (1). Collectively, currently available antifungals demonstrate activity against yeasts, molds, and dimorphic fungi (polyene drug class) and are used to treat invasive aspergillosis (azoles), invasive candidiasis including candidemia (echinocandins), or cryptococcal meningitis (flucytosine + polyenes). However, limitations due to lack of oral (PO) formulations (polyenes and echinocandins), nephrotoxicity (polyenes), drug-drug interactions (azoles), erratic exposure (azoles), toxicities (azoles), and bone marrow suppression (flucytosine) exist. Additionally, growing resistance among fungal pathogens necessitates the development of new antifungals (2).

Fosmanogepix (FMGX, PF-07842805, APX001, E1211), an orally bioavailable prodrug, has a novel mechanism of action and is a first-in-class “gepix” antifungal. It is metabolized into its active form, manogepix (MGX, APX001A, E1210) by systemic alkaline phosphatases. MGX selectively inhibits fungal glycosyl phosphatidyl inositol (GPI)-anchored wall protein transfer (Gwt1; and not the human ortholog, phosphatidylinositol glycan anchor biosynthesis class W (PIG-W) (3, 4), essential for glycosylphosphatidylinositol-anchored mannoprotein synthesis and maturation, thus blocking fungal growth and preventing host cell invasion (3, 5). *In vitro*, when compared with azoles (fluconazole, itraconazole, voriconazole), amphotericin B, and micafungin, MGX had lower or comparable minimum inhibitory concentration required for 90% inhibition (MIC_90_) against *Candida* spp. (except *Candida krusei*), *Aspergillus fumigatus*, *Cryptococcus* spp., *Coccidioides* spp., rare molds such as *Fusarium solani* and other black molds, and azole- and echinocandin-resistant isolates (6, 7). In murine models of disseminated candidiasis (*Candida albicans*), pulmonary aspergillosis (*Aspergillus fumigatus* or *Aspergillus flavus*), and disseminated fusariosis (*Fusarium solani*), PO FMGX demonstrated >80% survival for 2 weeks at doses of 2.5–25 mg/kg/day (8).

In two phase 1 studies of FMGX in healthy volunteers (NCT02956499 and NCT02957929) (9), a maximum intravenous (IV) dose of 600 mg (3-h infusion) and PO dose of 1,000 mg were administered for 14 days. These FMGX doses were safe and well-tolerated with a linear pharmacokinetics (PK) profile and no dose-limiting toxicities; therefore, the maximum tolerated dose (MTD) could not be determined (10, 11). Here, combined results from two phase 1, placebo (PBO)-controlled studies conducted in healthy participants to assess the safety, tolerability, and PK of multiple IV and PO doses (including IV-to-oral switch) of FMGX administered over 42 days [study 1 (105)] and multiple IV doses of FMGX (to assess the MTD) administered over 7 days [study 2 (106)] are presented.

## MATERIALS AND METHODS

### Study design

Two randomized, double-blind, PBO-controlled, multiple-dose phase 1 FMGX studies (study 1: EudraCT number: 2018-001351-12 and study 2: EudraCT number: 2018-002041-11) were conducted in healthy participants (randomized 3:1 to FMGX: placebo). Both studies were sponsored by Amplyx Pharmaceuticals (now a subsidiary of Pfizer, Inc.) and conducted by ICON (former PRA Health Sciences), Groningen, the Netherlands. Participation was voluntary and voluntary withdrawal was permitted at any time, regardless of reason.

### Key eligibility criteria: study 1 and study 2

Medically fit, healthy adults (age: study 1—18–65 years; study 2—18–55 years; body mass index: 18.0–30.0 kg/m^2^) with biochemical parameters within normal limits were eligible. Participants with uncontrolled or active major systemic disease and acute/chronic infection and history or presence of malignancy within the past year were excluded, as were females with childbearing potential unwilling to use two birth control methods. Those participants with significant/acute illness within 5 days prior to first FMGX administration or who were part of another investigational drug study within 60 days prior to the first FMGX administration were not eligible.

### Study design

#### Study 1

Study 1 was conducted from May to September 2018 and included a 42-day treatment period consisting of IV dosing (days 1–7) followed by an oral switch (days 8–42). Twelve participants were randomized either to placebo or FMGX [loading dose of 1,000 mg infused over 3 h twice daily (BID) on day 1 (9 h apart); 600 mg infused once daily (QD) between day 2 and day 7; and 800 mg oral tablets QD taken from days 8 to 42]. Screening was conducted within 28 days prior to the first dose of FMGX and participants were admitted to the clinical research center (CRC) 1 day prior to the first dose of FMGX. After discharge on day 28, participants continued taking FMGX daily until day 41. Participants took the last dose of FMGX at the CRC on day 42. Ambulatory visits to the CRC were planned for day 31, days 34 and 35 (one-night stay), and day 38. Participants also maintained a record of the frequency and amount of FMGX taken, including details of their well-being (days 29–34 and days 36–41). Participants returned to the CRC on day 41 and were discharged on day 44, followed by an ambulatory visit on day 49. The final follow-up visit was conducted at the CRC on day 56.

#### Study 2

**Fig 1:**
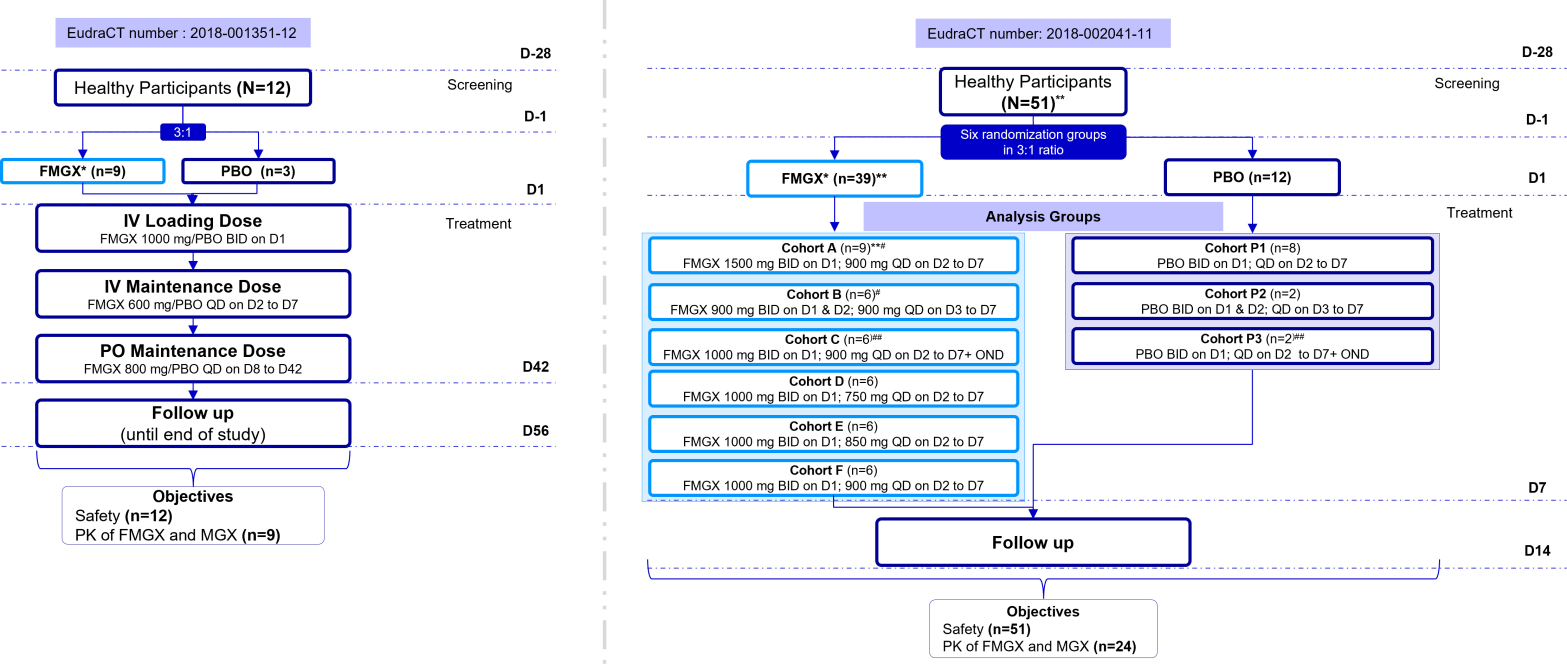
Study design. *IV FMGX was given as 3-h infusion; **including three replaced participants receiving FMGX in cohort A; #A sentinel dosing design was applied to cohorts 1 and 2; first 2 participants of each cohort (one receiving FMGX and one PBO) received 7 days of dosing prior to remaining participants in the cohort. If found safe, remaining (five receiving FMGX and one PBO) were dosed; ##OND was given as a tablet of 8 mg. BID, twice a day; D, day; FMGX, fosmanogepix; IV, intravenous; MGX, manogepix; OND, ondansetron; PBO, placebo; PK, pharmacokinetics; PO, oral; QD, once a day.

Study 2 was conducted from July 2018 to March 2019 and enrolled 51 participants (including three replacement participants) in six multiple dose cohorts (cohorts A–F, shown in Fig. 1) with eight participants each. Screening was completed within 28 days prior to first FMGX dose. After admission to the study center on day -1, the first dose of FMGX was administered on day 1. Thereafter, dosing continued for 7 consecutive days, following which participants were discharged on day 9 after completion of assessments. Participants visited the study center on day 11 for an additional ambulatory visit with subsequent follow-up assessments performed on day 14.

Sentinel dosing was applied to cohorts A and B and was to be applied to the remaining four cohorts (cohorts C to F) if dose escalation occurred. The first two participants of cohorts A and B received 7 days of dosing (randomized 1:1 to FMGX:placebo; prior to the remaining participants in the same cohort). After the Safety Review Committee (SRC; consisting of the Investigator and medical monitor) reviewed 7-day data from the first two participants, the remaining six were randomized (FMGX:placebo = 5:1). Cohorts C–F did not have a sentinel dosing design (no dose escalation during maintenance). Due to the poor tolerability of the treatment regimen administered to cohort B, dose escalation was not recommended for cohort C and the loading dose was administered on day 1 only. Additionally, oral ondansetron was included prior to the start of cohort C infusions to reduce the incidence and severity of treatment-emergent adverse events (TEAEs) (nausea and vomiting). For cohorts D–F, the SRC recommended prophylactic ondansetron only if deemed necessary by the Investigator. However, oral ondansetron was not administered in any other cohort.

Participants were assigned either to placebo or FMGX (3-h IV infusions) with loading doses administered BID on day 1 (and on day 2 in cohort B; 9 h apart) followed by maintenance doses administered QD on days 2–7 (days 3–7 in cohort B). Dosing schedules (six multiple dose cohorts) were as follows: cohorts A and B, loading doses of 1,500 mg and 900 mg, respectively, and maintenance dose of 900 mg; cohorts C, D, E, and F, loading dose of 1,000 mg and maintenance doses of 900 mg, 750 mg, 850 mg, and 900 mg, respectively. In cohort C, 8 mg oral ondansetron was administered with a meal approximately 2 h prior to starting each infusion. No ondansetron was administered in cohorts D–F (Fig. 1).

### Study assessments

#### PK assessment

Blood samples (4 mL) were taken at various time points for plasma PK analysis, and PK parameters for FMGX and MGX were calculated by non-compartmental analysis from plasma concentration time data.

##### Study 1

The PK predose blood sample was taken within 60 minutes before initiation of IV or PO doses. The 16-, 18-, and 24-h post-day 1 dose samples were collected on day 2 (24 h post-day 1 sample coincided with day 2 predose). PK blood samples were also collected after administration of the following doses: 24 h post-day 4 (coincided with day 5 predose); 16 and 24 h post-day 7 on day 8, post-day 8 on day 9, and post-day 14 on day 15 (coincided with predose samples on days 8, 9, and 15 respectively); 16-, 24-, and 36-h post-day 42 on day 43 (coincided with day 43 pre-sample); and 48-, 168-, and 336-h post-day 42 on days 44 (coincided with day 44 pre-sample), 49, and 56.

##### Study 2

For cohorts A and C–F, the 16-, 18-, and 24-h post-day 1 dose samples were collected predose on day 2 (for cohort B, 16-, 18-, and 24-h post-days 1 and 2 dose samples were collected predose on days 2 and 3, respectively). PK blood samples were also collected subsequent to the following doses: 24-h post-day 4 dose on day 5 (= day 5 predose); 16-, 24-, and 36-h post-day 7 on day 8; 48-h post-day 7 on day 9; day 11 sample (day 11 visit); and day 14 sample (day 14 visit).

### Safety and tolerability assessments

TEAEs, vital signs, clinical laboratory parameters, and body weight were recorded, and physical examinations, 12-lead electrocardiograms, and neurological examinations were performed. TEAEs were recorded from first admission through last follow-up and identified as events not present prior to FMGX administration or those that worsened (in severity or frequency) following FMGX exposure. All TEAEs were classified based on Medical Dictionary for Regulatory Activities (MedDRA) Version 21 and their severity was rated using Common Terminology Criteria for Adverse Events (CTCAE) Version 5. Those TEAEs which were assessed as “possibly related” or “related” were reported as related to FMGX.

The SRC could recommend that the sponsor suspend the study based on predefined dose-escalation stopping rules.

### Bioanalytical assessment

Analyses of FMGX and MGX in plasma samples were performed using validated liquid chromatography-tandem mass spectrometry (LC-MS/MS) (ICON Bioanalytical Laboratory, former PRA Health Sciences, Assen, the Netherlands). Sample processing was performed by means of protein precipitation using sample volumes of 50.0 µL (FMGX) and 20.0 µL (MGX). Separation from potential interfering endogenous compounds/metabolites was achieved by high performance liquid chromatography (HPLC) using a Waters Xbridge C18 column at 25°C, and using 25 mM ammonium bicarbonate in water (pH = 9) as mobile phase A and acetonitrile as mobile phase B operating at a gradient with an initial flow rate of 1.00 mL/min. An API-5000 quadrupole mass spectrometer equipped with a turbo ion spray source was used for detection in positive ion mode. Quantification was based on multiple reaction monitoring of the transitions of FMGX/MGX and their internal standards. A linear calibration curve with a 1 /x^2^ weighing factor was used ranging from 0.500 to 1,000 ng/mL for FMGX and from 10.0 to 20,000 ng/mL for MGX. Due to rapid *in vivo* conversion of FMGX to MGX, the lower limit of quantification (LLOQ) for FMGX (0.50 ng/mL) was lower than for MGX (LLOQ: 10 ng/mL). Quality control samples were included in each run to monitor run performance.

### Statistical analysis

Prospective statistical power calculations were not used to determine sample sizes. Safety analysis population included participants who received at least one dose of FMGX or placebo and PK analysis population included all participants who received at least one dose of FMGX and with sufficient bioanalytical data to reliably estimate PK parameters.

Demographics, PK parameters, and safety data were summarized using descriptive statistics. Plasma PK parameters were estimated from concentration-time profiles using non-compartmental methods and compartmental modeling. Statistical analyses were performed using SAS version 9.4.

## RESULTS

### Baseline characteristics and disposition

#### Study 1: IV-infused/oral switch of FMGX

The demographics and baseline characteristics between FMGX and placebo arms were balanced (Table 1). Most participants were white males. A total of 24 participants were screened and 12 were enrolled, of whom nine received FMGX and three received placebo. Eleven participants completed the study. One participant withdrew due to personal reasons before study completion.

**TABLE 1:** Demographics and baseline characteristics (safety population)^*a*^^,*b*^

	Study 1 (IV infusion/oral switch)		Study 2 (multiple doses of IV infusion)								
	PBO (*N* = 3)	FMGX 1,000 mg and 600 mg IV; 800 mg oral (*N* = 9)	PBO			FMGX					
			PBO 1 (*N* = 8)	PBO 2 (*N* = 2)	PBO 3 (*N* = 2)	Cohort A 1,500/900 mg (*N* = 9)	Cohort B 900/900 mg (*N* = 6)	Cohort C 1,000/900 mg (*N* = 6)	Cohort D 1,000/750 mg (*N* = 6)	Cohort E 1,000/850 mg (*N* = 6)	Cohort F 1,000/900 mg (*N* = 6)
Sex (male, %)	67	100	25	0	0	22.2	0	16.7	33.3	50.0	16.7
Race (White/Black/Asian/Other, %)	67/0/33/0	78/11/0/11	87.5/0/12.5/0	100/0/0/0	100/0/0/0	66.7/0/0/33.3	100/0/0/0	83.3/0/0/16.7	100/0/0/0	83.3/0/16.7/0	83.3/16.7/0/0
Age, mean (SD) (years)	52 (11)	42 (18)	25 (4)	28 (3)	38 (15)	29 (13)	30 (11)	26 (6)	23 (2)	29 (3)	34 (11)
Weight, mean (SD) (kg)	74.8 (27.5)	79.4 (9.3)	64.7 (9.9)	65.8 (0.3)	67.1 (4.0)	68.7 (8.2)	74.8 (6.2)	67.7 (10.5)	70.6 (7.9)	71.6 (5.1)	73.2 (10.4)
Height, mean (SD) (cm)	170 (21)	179 (8)	167 (9)	174 (1)	174 (3)	169 (8)	174 (6)	173 (5)	174 (8)	172 (5)	172 (8)
BMI, mean (SD) (kg/m^2^)	25.4 (4.1)	24.9 (3.9)	23.1 (1.7)	21.9 (0.3)	22.2 (2.1)	24.0 (2.5)	24.7 (1.6)	22.5 (2.5)	23.3 (1.5)	24.2 (1.4)	24.7 (1.8)

^*a*^ Cohorts: PBO 1, PBO BID on day 1 and QD on days 2 to 7 IV infusion; PBO 2, PBO BID on days 1 and 2 and QD on days 3 to 7 IV infusion; PBO 3, PBO BID on day 1 and QD on days 2 to 7 IV infusion with ondansetron; A, 1,500 mg BID on day 1 and 900 mg QD on days 2 to 7 FMGX IV infusion; B, 900 mg BID on days 1 and 2 and 900 mg QD on days 3 to 7 FMGX IV infusion; C, 1,000 mg BID on day 1 and 900 mg QD on days 2 to 7 FMGX IV infusion with ondansetron; D, 1,000 mg BID on day 1 and 750 mg QD on days 2 to 7 FMGX IV infusion; E, 1,000 mg BID on day 1 and 850 mg QD on days 2 to 7 FMGX IV infusion; F, 1,000 mg BID on day 1 and 900 mg QD on days 2 to 7 FMGX IV infusion.

^*b*^ BID, twice daily; BMI, body mass index; FMGX, fosmanogepix; IV, intravenous; PBO, placebo; QD, once daily; SD, standard deviation.

#### Study 2: multiple IV doses of FMGX

Demographics and baseline characteristics between FMGX and PBO arms were balanced (Table 1). Most participants were white females. Overall, 51 participants were exposed to study treatment, of whom 39 received FMGX (including three replacement participants in cohort A) and 12 received placebo. A total of 34 participants completed the study. Five participants were withdrawn due to TEAEs (two in cohort A and one each in cohorts B, C, and E), 11 withdrew their consent since they considered FMGX not well-tolerated (one in cohort A, three in B, four in C, one in E and two while receiving PBO), and one was withdrawn due to bad venous access.

### Pharmacokinetics

#### Study 1: IV-infused/oral switch of FMGX

**Fig 2:**
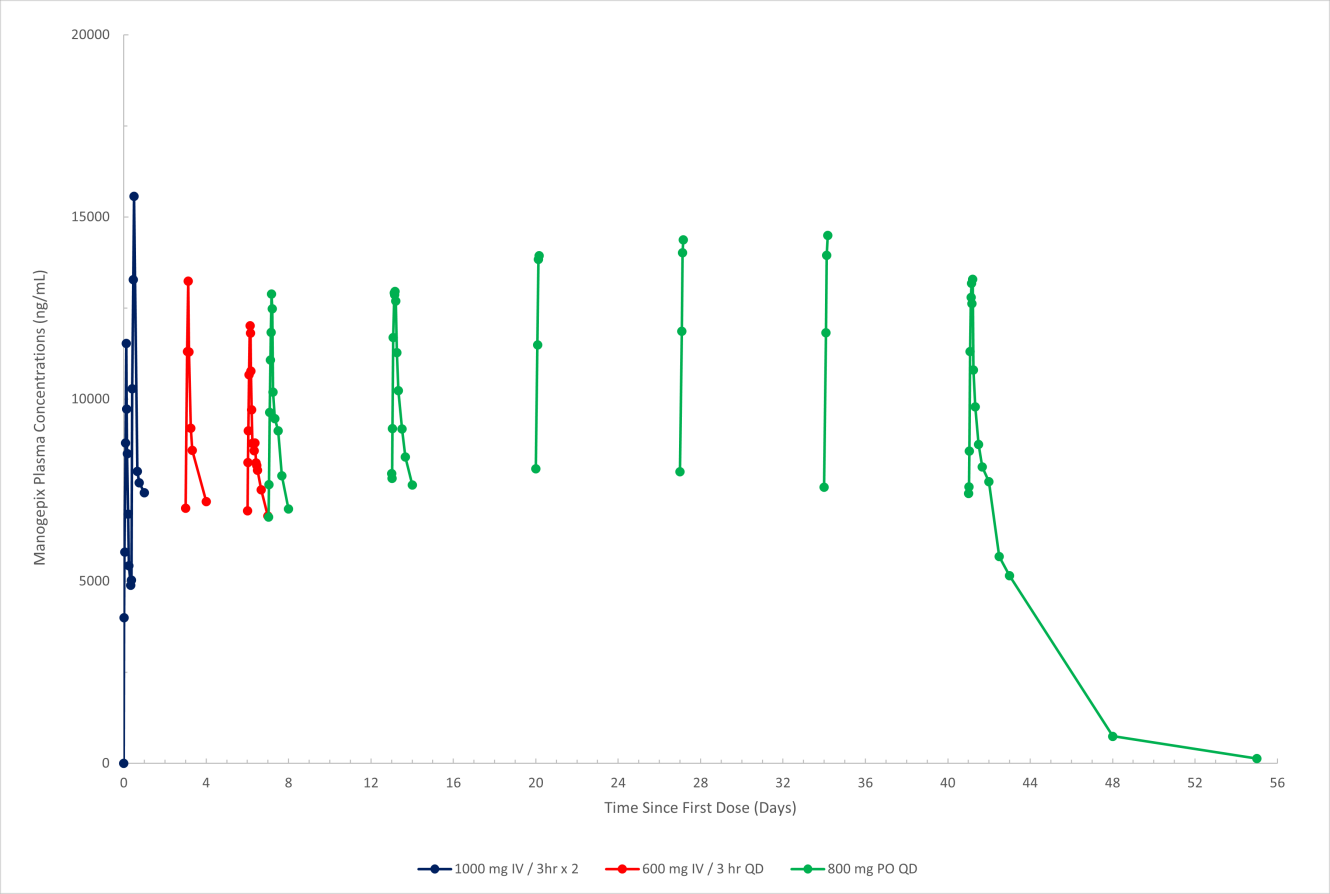
Geometric mean plasma concentrations of MGX after IV-infused/oral switch of FMGX. FMGX, fosmanogepix; hr, hours; IV, intravenous; MGX, manogepix; PO, oral; QD, once daily.

FMGX was rapidly converted to MGX after IV administration (Table S1), similar to observations from previous PK evaluations of FMGX (9). In the majority of participants, FMGX plasma concentrations were only quantifiable for 4–12 h after IV infusion on days 1–7 and were not quantifiable after oral administration due to first pass metabolism. Geometric mean plasma concentrations of MGX were higher after administration of two 1,000 mg IV FMGX loading doses (day 1) and remained relatively consistent from days 4 to 7 (600 mg IV). No decrease in MGX concentrations were observed after the switch from IV to oral FMGX (day 8; Table 2; Fig. 2). Slight increases in MGX plasma concentrations over days 8 to 21 were observed as steady-state was achieved. Thereafter, MGX concentrations were consistent through day 42 . Overall mean plasma MGX concentrations were generally comparable throughout the 42-day treatment period, indicating that steady-state concentrations were achieved by administration of two 1,000 mg IV loading doses within 24 h and maintained by daily 600 mg IV infusions through day 7 followed by switch to 800 mg daily oral doses of FMGX up to day 42.

**TABLE 2:** MGX pharmacokinetic parameters after IV-infused/oral switch of FMGX (study 1)^*a*^^,^^*b*^^,^^*c*^

Study 1, *N* = 9 Parameter	IV infusion			Oral					
	2 × 1,000 mg	600 mg		800 mg					
	Day 1	Day 4	Day 7	Day 8	Day 14	Day 21	Day 28	Day 35	Day 42
*C*_max_ (ng/mL)	15,564 [28.7]	13,237 [24.7]	12,526[17.7]	13,895 [13.8]	14,831 [14.5]	14,736[21.3]	15,708 [14.4]	15,722[13.2]	15,383 [17.6]
AUC_0-24_ (ng·h/mL)	197,821 [35.1]	201,881 [22.6]	194,912 [20.5]	208,036 [16.9]	223,610 [12.5]	–	–	–	224,285 [16.8]
t_½_ (h)	–	–	–	–	–	–	–	–	49.6 [39.3]
*T*_max_ (h)	12.0 [12.0–12.0]	3.05 [3.00–3.08]	3.03 [2.00–3.50]	4.00 [2.00–8.00]	3.02 [2.00–5.00]	4.00 [2.00–4.00]	4.00 [2.00–4.00]	4.00 [2.00–4.00]	3.50 [3.00–5.00]
CL (mL/h/kg)	–	–	39.0 [15.8]	–	–	–	–	–	34.6 [13.6]
Vz (L/kg)	–	–	–	–	–	–	–	–	2.47 [38.6]

^*a*^ Data are presented as geometric mean [geometric %CV].

^*b*^ Dash denotes that the parameter was not applicable to the study day.

^*c*^ AUC_0-24_, area under the concentration-time curve from time zero to 24 h postdose; AUC_0-t_, area under the concentration-time curve from zero to time t postdose; *C*_max_, maximum plasma concentration; %CV, percent coefficient of variation; CL, clearance; IV, intravenous; t_1/2_, terminal phase half-life; FMGX, fosmanogepix; MGX, manogepix; *T*_max_, time to maximum plasma concentration (*C*_max_); Vz, volume of distribution.

During the IV dosing period, the overall extent of exposure [measured by geometric mean area under the concentration-time curve from time zero to 24 h postdose (AUC_0-24_)] was generally comparable on day 1 (197,821 ng·h/mL), day 4 (201,881 ng·h/mL), and day 7 (194, 912 ng·h/mL). The highest geometric mean peak exposure (C_max_) occurred on day 1 (15,564 ng/mL) 3 h after the start of the second IV loading dose. On day 4 (13,237 ng/mL) and day 7 (12,526 ng/mL), geometric mean peak exposures were slightly lower than on day 1 and occurred at the end of the 3-h IV infusion (median *T*_max_ = 3.05 h and 3.03 h, respectively; Table 2; Fig. 2).

On day 8, after the switch from 600 mg IV (day 7) to 800 mg oral FMGX, there was an increase in the geometric mean *C*_max_ to 13,895 ng/mL. Subsequently, *C*_max_ increased beyond levels achieved during IV administration, ranging from 14,736 ng/mL to 15,708 ng/mL. The geometric mean AUC_0-24_ remained reasonably consistent with that from the IV phase [208,036 ng·h/mL on day 8 and 223,610 ng·h/mL and 224,285 ng·h/mL on days 14 and 42, respectively (Table 2)].

Median *T*_max_ values ranged from 3.02 to 4.0 h throughout the study with overlapping ranges, suggesting no apparent change due to multiple dosing. Geometric mean t½ was 49.6 h.

#### Study 2: multiple IV doses of FMGX

**Fig 3:**
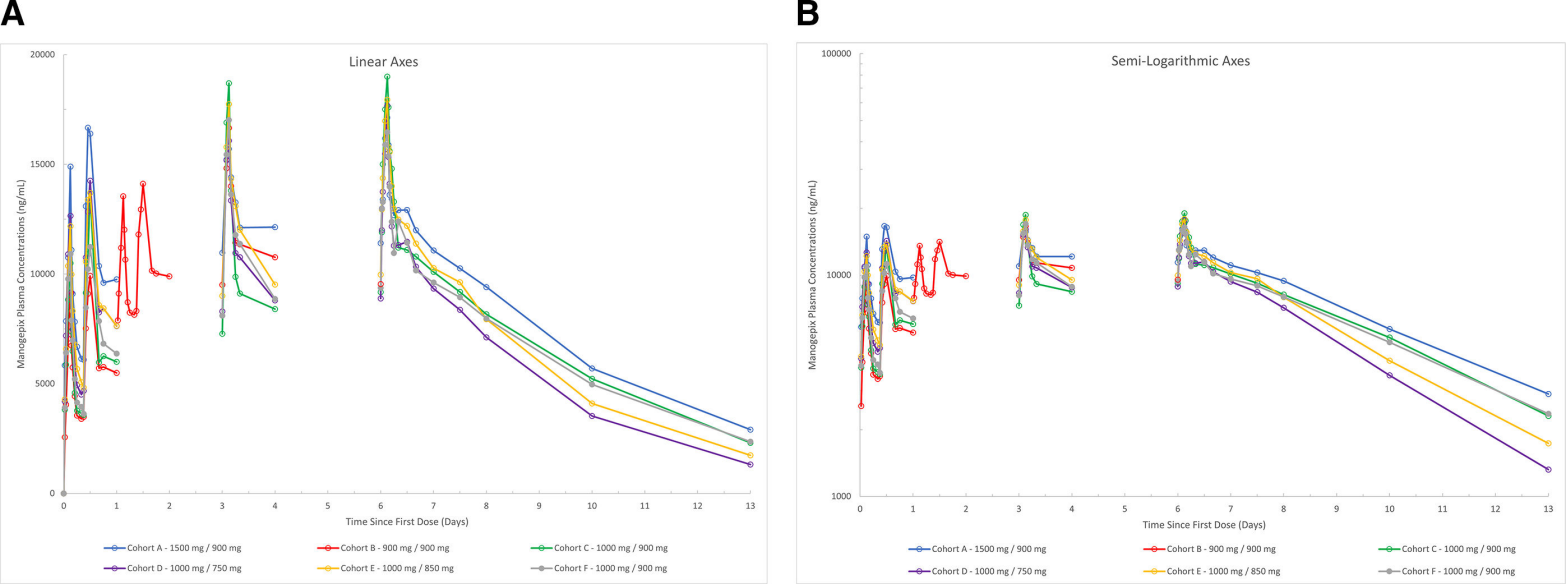
Geometric mean plasma concentrations of MGX after multiple doses of IV-infused FMGX—linear (left panel) and semilogarithmic (right panel) axes. IV, intravenous; FMGX, fosmanogepix; MGX, manogepix.

FMGX was rapidly converted to MGX after IV administration (Table S2), similar to observations from previous PK evaluations of FMGX with measurable FMGX concentrations available for only 4–24 h after IV infusions on days 1–7 (12). Geometric mean plasma concentrations of MGX on day 1 were highest for cohort A, with a loading regimen of 1,500 mg/3 h BID (9 h apart). Cohort B received a loading regimen of 900 mg/3 h BID (9 h apart) on days 1 and 2 and had a slightly different concentration profile from the other cohorts. Geometric mean *C*_max_ and AUC_0-24_ were lower in cohort B (10,571 ng/mL and 133,655 h·ng/mL, respectively) due to loading dose of 900 mg IV BID on days 1 and 2. Cohorts C through F all received a loading regimen of 1,000 mg/3 h BID (9 h apart) and had comparable profiles, as indicated by geometric mean *C*_max_ and AUC_0-24_ on day 1 (Table 3; Fig. 3). Consistent with the use of a loading regimen, steady state was achieved between days 2 and 4. All cohorts had similar geometric mean plasma concentrations during the maintenance period and similar PK parameters. The mean *C*_max_ and AUC_0-24_ were highest on day 1 (17,436 ng/mL and 240,839 h·ng/mL, respectively) and were comparable in cohorts C through F (12,316–14,255 ng/mL and 164,806–205,589 h·ng/mL, respectively).

**TABLE 3:** MGX pharmacokinetic parameters after multiple doses of IV-infused FMGX (study 2)^*c*^^,^^*d*^^,^^*e*^

Study 2	N	Day 1			Day 2			Day 4			Day 7					
		*C*_max_ (ng/mL)	AUC_0-24_ (ng·h/ mL)	*T*_max_ (h)	*C*_max_ (ng/mL)	AUC_0-24_ (ng·h/ mL)	*T*_max_ (h)	*C*_max_ (ng/mL)	AUC_0-24_ (ng·h/ mL)	*T*_max_ (h)	*C*_max_ (ng/mL)	AUC_0-24_ (ng·h/ mL)	*T*_max_ (h)	T_½_ (h)	CL (mL/h/kg)	Vz (L/kg)
Cohort A 1,500/900 mg	5	17,436 [7.88]	240,839 [9.77]	11.0 [11.0–12.2]	–	–	–	16,193 [8.59]	294,808 [9.03]	3.00 [2.00–3.92]	17,178 [9.53]	305,887 [11.6]	3.03 [2.92–3.50]	67.9 [31.0]^*a*^	32.1 [15.3]	3.42 [23.4]^*a*^
Cohort B 900/900 mg	2	10,571 [34.5]	133,655 [32.6]	7.50 [3.00–12.0]	13,517 [15.8]	236,001 [22.2]	12.0 [12.0–12.0]	16,733 [12.71]	280,660 [14.5]	3.04 [3.00–3.08]	14,892 [4.75]	270,745 [7.87]	3.01 [3.00–3.02]	89.0^*b*^	31.6 [4.52]	4.18^*b*^
Cohort C 1,000/900 mg	1	13,700	164,806	12.0 [12.0–12.0]	–	–	–	18,700	238,666	3.00 [3.00–3.00]	19,000	289,695	3.00 [3.00–3.00]	69.0	33.5	3.34
Cohort D 1,000/750 mg	6	14,255 [20.1]	202,026 [19.7]	12.0 [12.0–12.0]	–	–	–	16,139 [15.0]	254,095 [12.5]	3.02 [2.00–3.22]	16,859 [13.6]	273,327 [12.0]	3.01 [2.00–3.03]	51.7 [24.5]	29.9 [16.9]	2.23 [41.4]
Cohort E 1,000/850 mg	4	14,150 [33.6]	205,589 [26.1]	12.1 [11.0–12.1]	–	–	–	17,740 [15.6]	279,977 [13.8]	3.00 [3.00–3.02]	18,017 [14.00]	300,755 [9.31]	2.52 [2.00–3.08]	58.8 [46.0]	31.3 [15.3]	2.66 [43.4]
Cohort F 1,000/900 mg	6	12,316 [19.4]	172,244 [15.6]	12.0 [3.03–16.0]	–	–	–	17,018 [6.87]	265,124 [10.0]	3.01 [2.98–3.32]	16,850 [11.0]	263,509 [15.1]	2.98 [0.00–8.00]	75.1 [41.3]	36.0 [15.2]	3.90 [51.7]

^*a*^ *n* = 3.

^*b*^ *n* = 1.

^*c*^ Data are presented as geometric mean [geometric %CV] except for *T*_max_ for which the median [range] is reported.

^*d*^ Dash denotes that the parameter was not applicable to the study day.

^*e*^ AUC_0-24_, area under the concentration-time curve from time zero to 24 h postdose; AUC_0-t_, area under the concentration-time curve from zero to time t postdose; *C*_max_, maximum plasma concentration; %CV, percent coefficient of variation; IV, intravenous; FMGX, fosmanogepix; MGX, manogepix; CL, clearance; t_1/2_, terminal phase half-life; *T*_max_, time to maximum plasma concentration (*C*_max_); Vz, volume of distribution.

While two cohorts (D and E) had maintenance doses of 750 mg and 850 mg, respectively, exposure differences from the 900 mg maintenance doses used for the other four cohorts were minor and did not mirror the dose differences of 17% and 6%, respectively.

For all maintenance doses, the same range of variability was observed for PK parameters. Geometric mean plasma concentrations, *C*_max_, and AUC were comparable regardless of maintenance dose (Table 3; Fig. 3).

### Safety and tolerability

#### Study 1: IV-infused/oral switch of FMGX

All doses of FMGX administered during this study were safe and well-tolerated. No deaths, serious adverse events (SAEs), or withdrawals due to TEAEs were reported. A total of 12 participants reported 65 TEAEs, of which one occurrence of mild somnolence during the oral dosing period was considered related to FMGX treatment.

Eight participants (89%) reported 31 TEAEs during treatment with FMGX IV infusion (during the first 7 days) and seven participants (78%) reported 25 TEAEs during treatment with FMGX oral tablets (from day 8 onward; over the next 35 days of treatment). Two participants (67%) reported five TEAEs during treatment with placebo IV infusion and one participant (33%) reported four TEAEs during treatment with placebo oral tablets. The majority of TEAEs were mild, except for two moderate TEAEs of backache (reported during treatment with FMGX IV infusion) and headache (reported during treatment with FMGX oral tablets). Both events were considered unrelated to FMGX administration and resolved with paracetamol treatment (Table 4; Table S3).

**TABLE 4:** TEAEs for IV-infused/oral switch of FMGX and multiple doses of IV-infused FMGX^*d*^^,^^*e*^^,^^*f*^

A. Study 1 (IV-infused/oral switch of FMGX)				
TEAEs^*a*^ E/n (%)	PBO		FMGX	
	IV infusion (*N* = 3)	Oral tablets (*N* = 3)	IV infusion 2 × 1,000 and 600 mg (*N* = 9)	Oral tablets 800 mg (*N* = 9)
All TEAEs	5/2 (67)	4/1 (33)	31/8 (89)	25/7 (78)
FMGX-related TEAEs	0/0 (0)	0/0 (0)	0/0 (0)	1/1 (11)
Nervous system disorders (somnolence)	0/0 (0)	0/0 (0)	0/0 (0)	1/1 (11)
TEAEs by severity				
Mild	5/2 (67)	4/1 (33)	30/8 (89)	24/7 (78)
Moderate	0/0 (0)	0/0 (0)	1/1 (11)	1/1 (11)
Musculoskeletal and connective tissue disorders (backache)	0/0 (0)	0/0 (0)	1/1 (11)	0/0 (0)
Nervous system disorders (headache)	0/0 (0)	0/0 (0)	0/0 (0)	1/1 (11)
Deaths and other SAEs	0/0 (0)	0/0 (0)	0/0 (0)	0/0 (0)
Withdrawn due to TEAEs	0/0 (0)	0/0 (0)	0/0 (0)	0/0 (0)

B. Study 2 (multiple doses of IV-infused FMGX)									
TEAEs^*a*^ E/n (%)	PBO			FMGX					
	PBO 1 (*N* = 8)	PBO 2 (*N* = 2)	PBO 3 (*N* = 2)	Cohort A 1,500/900 mg (*N* = 9)	Cohort B 900/900 mg (*N* = 6)	Cohort C 1,000/900 mg (*N* = 6)	Cohort D 1,000/750 mg (*N* = 6)	Cohort E 1,000/850 mg (*N* = 6)	Cohort F 1,000/900 mg (*N* = 6)
All TEAEs	40/8 (100)	7/2 (100)	23/2 (100)	67/9 (100)	41/6 (100)	51/6 (100)	47/6 (100)	40/6 (100)	41/6 (100)
FMGX-related TEAEs^*b*^	15/5 (62.5)	2/1 (50.0)	6/1 (50.0)	37/8 (88.9)	29/6 (100)	27/5 (83.3)	34/5 (83.3)	20/5 (83.3)	19/6 (100)
Nervous system disorders	6/3 (37.5)	1/1 (50.0)	2/1 (50.0)	14/6 (66.7)	8/6 (100)	19/5 (83.3)	15/4 (66.7)	11/5 (83.3)	14/6 (100)
Gastrointestinal disorders	1/1 (12.5)	0/0 (0)	4/1 (50.0)	17/8 (88.9)	15/6 (100)	8/4 (66.7)	9/5 (83.3)	3/2 (33.3)	2/2 (33.3)
General disorders and administration site conditions	4/2 (25.0)	1/1 (50.0)	0/0 (0)	5/3 (33.3)	5/4 (66.7)	0/0 (0)	6/4 (66.7)	4/2 (33.3)	1/1 (16.7)
Musculoskeletal and connective tissue disorders	0/0 (0)	0/0 (0)	0/0 (0)	1/1 (11.1)	1/1 (16.7)	0/0 (0)	0/0 (0)	0/0 (0)	1/1 (16.7)
Eye disorders	1/1 (12.5)	0/0 (0)	0/0 (0)	0/0 (0)	0/0 (0)	0/0 (0)	0/0 (0)	1/1 (16.7)	0/0 (0)
Respiratory, thoracic, and mediastinal disorders	2/1 (12.5)	0/0 (0)	0/0 (0)	0/0 (0)	0/0 (0)	0/0 (0)	1/1 (16.7)	0/0 (0)	0/0 (0)
Vascular disorders	0/0 (0)	0/0 (0)	0/0 (0)	0/0 (0)	0/0 (0)	0/0 (0)	1/1 (16.7)	1/1 (16.7)	0/0 (0)
Injury, poisoning, and procedural complications	0/0 (0)	0/0 (0)	0/0 (0)	0/0 (0)	0/0 (0)	0/0 (0)	0/0 (0)	0/0 (0)	1/1 (16.7)
Metabolism and nutrition disorders	0/0 (0)	0/0 (0)	0/0 (0)	0/0 (0)	0/0 (0)	0/0 (0)	1/1 (16.7)	0/0 (0)	0/0 (0)
Psychiatric disorders	0/0 (0)	0/0 (0)	0/0 (0)	0/0 (0)	0/0 (0)	0/0 (0)	1/1 (16.7)	0/0 (0)	0/0 (0)
Skin and subcutaneous tissue disorders	1/1 (12.5)	0/0 (0)	0/0 (0)	0/0 (0)	0/0 (0)	0/0 (0)	0/0 (0)	0/0 (0)	0/0 (0)
TEAEs by severity									
Mild	39/7 (87.5)	6/1 (50.0)	23/2 (100)	52/1 (11.1)	21/1 (16.7)	50/5 (83.3)	43/4 (66.7)	37/4 (66.7)	37/3 (50.0)
Moderate^*c*^	1/1 (12.5)	1/1 (50.0)	0/0 (0)	15/8 (88.9)	20/5 (83.3)	1/1 (16.7)	4/2 (33.3)	3/2 (33.3)	3/2 (33.3)
Gastrointestinal disorders	0/0 (0)	0/0 (0)	0/0 (0)	8/6 (66.7)	11/5 (83.3)	0/0 (0)	2/1 (16.7)	0/0 (0)	1/1 (16.7)
Nervous system disorders	0/0 (0)	0/0 (0)	0/0 (0)	3/2 (22.2)	5/5 (83.3)	1/1 (16.7)	1/1 (16.7)	1/1 (16.7)	1/1 (16.7)
General disorders and administration site conditions	0/0 (0)	0/0 (0)	0/0 (0)	3/3 (33.3)	2/2 (33.3)	0/0 (0)	1/1 (16.7)	2/1 (16.7)	1/1 (16.7)
Musculoskeletal and connective tissue disorders	0/0 (0)	1/1 (50.0)	0/0 (0)	1/1 (11.1)	1/1 (16.7)	0/0 (0)	0/0 (0)	0/0 (0)	0/0 (0)
Infections and infestations	0/0 (0)	0/0 (0)	0/0 (0)	0/0 (0)	1/1 (16.7)	0/0 (0)	0/0 (0)	0/0 (0)	0/0 (0)
Reproductive system and breast disorders	1/1 (12.5)	0/0 (0)	0/0 (0)	0/0 (0)	0/0 (0)	0/0 (0)	0/0 (0)	0/0 (0)	0/0 (0)
Severe^*c*^	0/0 (0)	0/0 (0)	0/0 (0)	0/0 (0)	0/0 (0)	0/0 (0)	0/0 (0)	0/0 (0)	1/1 (16.7)
Nervous system disorders (Syncope)	0/0 (0)	0/0 (0)	0/0 (0)	0/0 (0)	0/0 (0)	0/0 (0)	0/0 (0)	0/0 (0)	1/1 (16.7)
Deaths and other SAEs	0/0 (0)	0/0 (0)	0/0 (0)	0/0 (0)	0/0 (0)	0/0 (0)	0/0 (0)	0/0 (0)	0/0 (0)
Withdrawn due to TEAEs	0/0 (0)	0/0 (0)	0/0 (0)	2/2 (22.2)	3/1 (16.7)	1/1 (16.7)	0/0 (0)	1/1 (16.7)	0/0 (0)
FMGX-related	0/0 (0)	0/0 (0)	0/0 (0)	0/0 (0)	3/1 (16.7)	0/0 (0)	0/0 (0)	0/0 (0)	0/0 (0)

^*a*^ TEAEs were classified according to MedDRA Version 21.0. Participants were counted once for multiple occurrences of a specific MedDRA Preferred Term.

^*b*^ TEAEs by PT described in Table S2.

^*c*^ TEAEs by PT described in Table S3.

^*d*^ Note: Participants with multiple events were counted under the category of their highest severity (TEAEs by severity) or most FMGX-related event (TEAEs by relationship to FMGX).

^*e*^ Cohorts: PBO 1, PBO BID on day 1 and QD on days 2 to 7 IV infusion; PBO 2, PBO BID on days 1 and 2 and QD on days 3 to 7 IV infusion; PBO 3, PBO BID on day 1 and QD on days 2 to 7 IV infusion with ondansetron; A, 1,500 mg BID on day 1 and 900 mg QD on days 2 to 7 FMGX IV infusion; B, 900 mg BID on days 1 and 2 and 900 mg QD on days 3 to 7 FMGX IV infusion; C, 1,000 mg BID on day 1 and 900 mg QD on days 2 to 7 FMGX IV infusion with ondansetron; D, 1,000 mg BID on day 1 and 750 mg QD on days 2 to 7 FMGX IV infusion; E, 1,000 mg BID on day 1 and 850 mg QD on days 2 to 7 FMGX IV infusion; F, 1,000 mg BID on day 1 and 900 mg QD on days 2 to 7 FMGX IV infusion.

^*f*^ %, percentage of the total number of participants per treatment that experienced the AEs ([n/N]*100); BID, twice daily; E, number of times the AEs occurred; FMGX, fosmanogepix; IV, intravenous; N, number of participants exposed; n, number of participants that experienced TEAEs; PBO, placebo; PT, preferred term; QD, once daily; SAE, serious adverse events; TEAEs, treatment-emergent adverse events.

No clinically relevant laboratory investigations, vital signs, electrocardiogram (ECG), physical examination, neurological examination, or body weight findings were reported.

Two participants experienced events of elevated alanine aminotransferase (ALT) levels, first apparent during week 5 of FMGX administration with maximum ALT value 150 IU/L (day 41) in one participant and 194 IU/L (day 42) in the other i.e., less than three times the upper limit of normal (68 IU/L). Minor changes (less than two times upper limit of normal) were also observed in aspartate aminotransferase (AST) levels in these participants. Both events occurred after discharge from the CRC. These lab values returned to within normal ranges approximately 20 days after onset. Both events were not considered clinically significant and were not captured as AEs.

#### Study 2: multiple IV doses of FMGX

All doses of FMGX administered during the study were considered safe and no SAEs or deaths were reported. However, not all doses of FMGX administered during the study were considered well-tolerated. Although no dose-limiting toxicities were reported and no safety test results met any of the *a priori* rules that prevented dose escalation, headache and nausea were reported frequently by most participants in cohorts A through C. Therefore, no maintenance dose escalations occurred above levels tested in cohort B.

Two treatment regimens were not well-tolerated, including a loading dose of 1,500 mg BID FMGX IV on day 1 followed by a maintenance dose of 900 mg QD on days 2–7 (cohort A) and a loading dose of 900 mg BID FMGX IV on days 1 and 2 followed by a maintenance dose of 900 mg QD on days 3–7 (cohort B). Concomitant oral ondansetron was added to cohort C; however, TEAEs (headache and nausea) continued to be reported. Overall, 16 participants were either withdrawn (*n* = 5) or withdrew consent (*n* = 11) due to tolerability issues. Of the five participants that were withdrawn from the study by the investigator, two were withdrawn from cohort A and one each from cohorts B, C, and E (Table 4). The three treatment regimens that were considered relatively well-tolerated were a loading dose of 1,000 mg BID on day 1 followed by a maintenance dose of either 750 mg QD (cohort D), 850 mg QD (cohort E), or 900 mg QD on days 2–7 (cohort F).

Of a total of 357 TEAEs reported in all 51 participants, 308 were mild and 1 was severe. Twenty-two participants reported 48 moderate TEAEs. The percentage of participants reporting moderate TEAEs and the number of moderate TEAEs were higher in cohort A [15 TEAEs in eight (88.9%) participants] and cohort B [20 TEAEs in five (83.3%) participants] versus other cohorts (Table 4). The most frequently reported TEAEs by preferred term were headache, nausea, catheter site pain, dizziness, vomiting, abdominal pain, somnolence, fatigue, infusion site irritation, infusion site pain, and back pain (Tables S4 and S5). Syncope (always coded as a severe event) occurred in one patient at midnight (approximately 17 h after receiving FMGX) and was considered likely to be FMGX-related. This event was not reported as a SAE and no FMGX dose changes were required. The participant recovered without treatment and completed the study (Table 4).

## DISCUSSION

Two phase 1, randomized, double-blind, PBO-controlled studies were conducted in healthy participants to assess the safety, tolerability, and PK of multiple IV and PO doses of FMGX over an extended duration of 42 days (study 1) and multiple IV doses of FMGX (maximum loading dose of 1,500 mg BID) over 7 days (study 2).

The results of study 1 indicated that treatment with FMGX IV loading dose of 1,000 mg BID followed by IV maintenance dose of 600 mg (3-h infusions) QD and 800 mg QD PO over 42 days was safe and well-tolerated. Comparable geometric mean plasma MGX concentrations were observed throughout and maintained during the switch from IV to oral treatment. Most TEAEs (63/65) were mild (of which only one event of somnolence was considered FMGX-related), transient, and did not require any intervention. Two moderate TEAEs, one each of backache and headache occurred. One event each of AST and ALT elevation occurred while participants were taking FMGX at home; neither was considered to be clinically significant. These findings support the duration and provide a detailed PK profile of the treatment regimen assessed in the phase 2 efficacy and safety studies of FMGX for the treatment of candidemia and/or invasive candidiasis caused by *Candida auris* (NCT04148287) (12) and invasive aspergillosis and other rare molds (NCT04240886) (6, 13, 14).

The results of study 2 indicated that treatment with FMGX IV doses (loading doses BID/maintenance doses QD; 3 hr infusion) of 1,500/900 mg (cohort A), 900/900 mg (cohort B), and 1,000/900 mg (with ondansetron; cohort C) were not well-tolerated, with most participants reporting nausea and vomiting (TEAEs) despite concomitant ondansetron (cohort C). FMGX IV doses (loading doses BID/maintenance doses QD; 3-h infusions) of 1,000/750 mg (cohort D), 1,000/850 mg (cohort E), and 1,000/900 mg (without ondansetron; cohort F) were relatively better tolerated compared with treatment regimens with higher loading doses assessed in the same study. Geometric mean plasma MGX concentrations on day 1 were found to be highest for cohort A and were comparable for cohorts C, D, E, and F. Results from study 2 [FMGX at IV (loading dose/maintenance dose) of 1,500/900, 900/900, and 1,000/900 mg] indicated that the tolerability of higher IV loading doses and/or IV maintenance doses were less favorable. One severe event of syncope occurred in a single patient more than 3 h after the IV dose of FMGX and after the patient returned home from the CRC.

IFDs caused by *Candida* spp., *Aspergillus* spp., and invasive rare molds require treatment with high-dose antifungals over a prolonged duration (15, 16). In studies of other antifungals, a combination of front-loading and maintenance doses has been assessed, with the objective of achieving higher blood plasma concentrations and target efficacy levels (2). From the assessments described herein, a loading dose of 1,000 mg FMGX IV BID followed by lower IV and oral maintenance doses (600 mg–800 mg) were well-tolerated. When higher FMGX loading and maintenance doses were administered, participants experienced an increased incidence of headache and nausea (known FMGX-related AEs), resulting in discontinuations due to tolerability issues. From our current evaluations, ondansetron did not improve nausea and vomiting after FMGX administration. Similar to other antifungals, gastrointestinal disturbances are commonly occurring TEAEs at higher FMGX doses (2, 17).

The data presented in this manuscript are from two separate single-center studies, conducted in healthy volunteers with a relatively small sample size. Safety, tolerability, and PK data from healthy volunteers may be different in patients with life-threatening IFDs. Larger trials are planned in order to further evaluate FMGX as a treatment option for patients with IFDs.

In conclusion, results from study 1 (FMGX at IV loading dose of 1,000 mg followed by IV 600 mg and day 8 switch to PO 800 mg over a duration of 42 days) were encouraging. A comparable IV PK profile with drug exposures maintained after switching to oral dosing allows an opportunity to use PO formulations in outpatient clinical settings that require prolonged FMGX treatment. FMGX has also been evaluated in phase 2 studies for up to 14 days in non-neutropenic patients with candidemia (NCT03604705: IV 1,000 mg BID on day 1 followed by IV 600 mg and an optional switch to PO 700 mg) (18, 19) and for up to 42 days in patients with infections caused by *Candida auris*, *Aspergillus* spp., and other rare molds [NCT04240886 (14) and NCT04148287 (12): FMGX IV loading dose of 1,000 mg BID on day 1 followed by IV 600 mg and PO switch to 800 mg) (6, 13, 20) with encouraging results (21). IV loading dose (1,000 mg twice on day 1) with IV (600 mg QD) or oral (800 mg QD) maintenance doses are planned for evaluation in phase 3 trials (22).

## Data Availability

Upon request, and subject to review, Pfizer will provide the data that support the findings of this study. Subject to certain criteria, conditions and exceptions, Pfizer may also provide access to the related individual de-identified participant data. See https://www.pfizer.com/science/clinical-trials/trial-data-and-results for more information.

## SUPPLEMENTAL MATERIAL

Tables S1 to S5 aac.01455-23-s0001.docx Two PK tables (S1 and S2) and three safety tables (S3 to S5).

ASM does not own the copyrights to Supplemental Material that may be linked to, or accessed through, an article. The authors have granted ASM a non-exclusive, world-wide license to publish the Supplemental Material files. Please contact the corresponding author directly for reuse.
