# Supplementary material for: Pharmacokinetics, safety, and tolerability of fosmanogepix IV to oral switch and multiple IV doses in healthy participants
Source: Antimicrob Agents Chemother. 2024 Mar 29;68(5):e01455-23. doi: 10.1128/aac.01455-23 (PMC11064621; doi:10.1128/aac.01455-23)
Supplement: Tables S1 to S5 — Two PK tables (S1 and S2) and three safety tables (S3 to S5). [file aac.01455-23-s0001.docx]

# **Supplementary Tables:**

# **Table S1. FMGX Pharmacokinetic Parameters After IV/oral Switch**

| **Study 1, N=9**  **Parameters** | **IV Infusion** | | |
| --- | --- | --- | --- |
|  | **2 x 1000 mg** | **600 mg** | |
|  | **Day 1** | **Day 4** | **Day 7** |
| C_max_ (ng/mL) | 3,602 [43.4] | 1,377 [23.7] | 1,433 [16.1] |
| AUC_0-t_ (ng·hr/mL) | 23,584 [44.8] | 2,837 [28.4] | 3,709 [16.9] |
| AUC_0-24_ (ng·hr/mL) | - | - | 3,879 [10.8]^*^ |
| t_½_ (hr) | - | - | 0.341 [18.5]^*^ |
| T_max_ (hr) | 10.0 [1.00-12.0] | 2.00 [2.00-3.00] | 2.18 [1.42-2.34]^*^ |
| CL (mL/hr/kg) | - | - | 1,986 [13.7]^*^ |
| Vz (L/kg) | - | - | 0.978 [25.5]^*^ |

^*^n=8

Geometric mean [geometric %CV] (N) except for Tmax for which the median [Range] is reported. AUC_0-24_ = area under the concentration-time curve from time zero to 24 h postdose; AUC_0-t_ = area under the concentration-time curve from zero to time t postdose; C_max_ = maximum plasma concentration; %CV = percent coefficient of variation; FMGX = fosmanogepix; IV = intravenous; CL = Clearance; MGX = manogepix; t_1/2_ = terminal phase half-life; T_max_ = time to maximum plasma concentration (C_max_); Vz = volume of distribution. Dash denotes that the parameter was not applicable to the study day

# **Table S2. FMGX Pharmacokinetic Parameters After IV-infused FMGX Doses**

| **Study 2** | **n** | **Day 1** | | | **Day 2** | | | **Day 4** | | | **Day 7** | | |
| --- | --- | --- | --- | --- | --- | --- | --- | --- | --- | --- | --- | --- | --- |
|  |  | **C_max_**  **(ng/mL)** | **AUC_0-t_**  **(ng·hr/mL)** | **T_max_ (hr)** | **C_max_**  **(ng/mL)** | **AUC_0-t_**  **(ng·hr/mL)** | **T_max_ (hr)** | **C_max_**  **(ng/mL)** | **AUC_0-t_**  **(ng·hr/mL)** | **T_max_ (hr)** | **C_max_**  **(ng/mL)** | **AUC_0-t_**  **(ng·hr/mL)** | **T_max_ (hr)** |
| Cohort A  1500/900 mg | 5 | 5,052  [11.1] | 31,999  [16.1] | 2.02  [1.00–11.0] | - | - | - | 2,234  [8.17] | 5,082  [12.5] | 2.00  [2.00 – 2.92] | 2,392  [17.1] | 5,982  [27.8] | 1.92  [0.50–3.00] |
| Cohort B  900/900 mg | 2 | 2,719  [4.16] | 21,492  [6.00] | 6.50  [2.00–11.0] | 2,380  [9.22] | 13,821  [49.9] | 7.00  [2.00–12.0] | 1,929  [5.13] | 4,085  [27.6] | 2.03  [2.00–2.07] | 1,720  [3.29] | 4,539  [0.41] | 2.00  [2.00–2.00] |
| Cohort C  1000/900 mg | 1 | 3,030 | 23,654 | 3.00  [3.00–3.00] | - | - | - | 1,890 | 4,642 | 3.00  [3.00–3.00] | 2,850 | 7,396 | 3.00  [3.00–3.00] |
| Cohort D  1000/750 mg | 6 | 3,043  [20.7] | 20,936  [39.5] | 10.0  [1.00–11.0] | - | - | - | 1,925  [19.3] | 4,254  [14.8] | 2.00  [1.98–3.00] | 1,872  [15.2] | 4,897  [18.7] | 0.99  [0.50–1.02] |
| Cohort E  1000/850 mg | 4 | 3,562  [19.8] | 24,383  [23.9] | 11.0  [3.02–11.0] | - | - | - | 2,443  [42.4] | 5,303  [35.9] | 2.00  [2.00–3.00] | 2,180  [11.1] | 5,682  [19.7] | 2.08  [2.00–3.00] |
| Cohort F  1000/900 mg | 6 | 3,140  [13.5] | 22,331  [20.1] | 11.0  [1.00–12.0] | - | - | - | 2,336  [7.96] | 5,120  [15.0] | 2.00  [2.00–2.08] | 1,518  [88.1] | 4,041  [78.6] | 1.53  [0.00–3.00] |

Geometric mean [geometric %CV] (N) except for Tmax for which the median [Range] is reported. AUC_0-24_ = area under the concentration-time curve from time zero to 24 h postdose; AUC_0-t_ = area under the concentration-time curve from zero to time t postdose; C_max_ = maximum plasma concentration; %CV = percent coefficient of variation; FMGX = fosmanogepix; IV = intravenous; CL = Clearance; MGX = manogepix; t_1/2_ = terminal phase half-life; T_max_ = time to maximum plasma concentration (C_max_); Vz = volume of distribution. Dash denotes that the parameter was not applicable to the study day

# **Table S3. All TEAEs by SOC and PT for IV-infused/oral Switch of FMGX (Study 1)**

| **TEAEs by SOC and PT^*^  E/n (%)** | **PBO** | | **FMGX** | | **Total**  **(N=12)** |
| --- | --- | --- | --- | --- | --- |
|  | **IV Infusion**  **(N=3)** | **Oral Tablets**  **(N=3)** | **IV Infusion**  **2 x 1000 & 600 mg**  **(N=9)** | **Oral Tablets**  **800 mg**  **(N=9)** |  |
| Any TEAEs | 5/2 (67) | 4/1 (33) | 31/8 (89) | 25/7 (78)^†^ | 65/12 (100) |
| General disorders and administration site conditions | 3/1 (33) | 1/1 (33) | 13/8 (89) | 3/3 (33) | 20/11 (92) |
| Infusion site irritation | 1/1 (33) | 0/0 (0) | 6/5 (56) | 0/0 (0) | 7/6 (50) |
| Vessel puncture site reaction | 1/1 (33) | 1/1 (33) | 3/3 (33) | 1/1 (11) | 6/6 (50) |
| Catheter site hematoma | 0/0 (0) | 0/0 (0) | 0/0 (0) | 1/1 (11) | 1/1 (8) |
| Catheter site inflammation | 0/0 (0) | 0/0 (0) | 1/1 (11) | 0/0 (0) | 1/1 (8) |
| Fatigue | 0/0 (0) | 0/0 (0) | 0/0 (0) | 1/1 (11) | 1/1 (8) |
| Infusion site erythema | 0/0 (0) | 0/0 (0) | 1/1 (11) | 0/0 (0) | 1/1 (8) |
| Infusion site extravasation | 0/0 (0) | 0/0 (0) | 1/1 (11) | 0/0 (0) | 1/1 (8) |
| Infusion site reaction | 0/0 (0) | 0/0 (0) | 1/1 (11) | 0/0 (0) | 1/1 (8) |
| Peripheral swelling | 1/1 (33) | 0/0 (0) | 0/0 (0) | 0/0 (0) | 1/1 (8) |
| Nervous system disorders | 0/0 (0) | 1/1 (33) | 11/4 (44) | 15/6 (67)^†^ | 27/8 (67) |
| Headache | 0/0 (0) | 0/0 (0) | 5/3 (33) | 11/3 (33) | 16/4 (33) |
| Dizziness | 0/0 (0) | 0/0 (0) | 0/0 (0) | 2/2 (22) | 2/2 (17) |
| Somnolence | 0/0 (0) | 0/0 (0) | 1/1 (11) | 1/1 (11)^†^ | 2/2 (17) |
| Disturbance in attention | 0/0 (0) | 1/1 (33) | 0/0 (0) | 0/0 (0) | 1/1 (8) |
| Head discomfort | 0/0 (0) | 0/0 (0) | 3/1 (11) | 0/0 (0) | 3/1 (8) |
| Paresthesia | 0/0 (0) | 0/0 (0) | 0/0 (0) | 1/1 (11) | 1/1 (8) |
| Presyncope | 0/0 (0) | 0/0 (0) | 1/1 (11) | 0/0 (0) | 1/1 (8) |
| Tremor | 0/0 (0) | 0/0 (0) | 1/1 (11) | 0/0 (0) | 1/1 (8) |
| Skin and subcutaneous tissue disorders | 2/1 (33) | 1/1 (33) | 1/1 (11) | 2/2 (22) | 6/5 (42) |
| Dry skin | 0/0 (0) | 0/0 (0) | 1/1 (11) | 1/1 (11) | 2/2 (17) |
| Skin irritation | 2/1 (33) | 1/1 (33) | 0/0 (0) | 0/0 (0) | 3/2 (17) |
| Acne | 0/0 (0) | 0/0 (0) | 0/0 (0) | 1/1 (11) | 1/1 (8) |
| Musculoskeletal and connective tissue disorders | 0/0 (0) | 0/0 (0) | 2/2 (22) | 1/1 (11) | 3/3 (25) |
| Back pain | 0/0 (0) | 0/0 (0) | 2/2 (22) | 0/0 (0) | 2/2 (17) |
| Arthralgia | 0/0 (0) | 0/0 (0) | 0/0 (0) | 1/1 (11) | 1/1 (8) |
| Gastrointestinal disorders | 0/0 (0) | 0/0 (0) | 2/2 (22) | 2/1 (11) | 4/2 (17) |
| Nausea | 0/0 (0) | 0/0 (0) | 2/2 (22) | 1/1 (11) | 3/2 (17) |
| Diarrhea | 0/0 (0) | 0/0 (0) | 0/0 (0) | 1/1 (11) | 1/1 (8) |
| Infections and infestations | 0/0 (0) | 1/1 (33) | 0/0 (0) | 0/0 (0) | 1/1 (8) |
| Nasopharyngitis | 0/0 (0) | 1/1 (33) | 0/0 (0) | 0/0 (0) | 1/1 (8) |
| Injury, poisoning, and procedural complications | 0/0 (0) | 0/0 (0) | 1/1 (11) | 1/1 (11) | 2/1 (8) |
| Arthropod bite | 0/0 (0) | 0/0 (0) | 1/1 (11) | 0/0 (0) | 1/1 (8) |
| Limb injury | 0/0 (0) | 0/0 (0) | 0/0 (0) | 1/1 (11) | 1/1 (8) |
| Metabolism and nutrition disorders | 0/0 (0) | 0/0 (0) | 0/0 (0) | 1/1 (11) | 1/1 (8) |
| Decreased appetite | 0/0 (0) | 0/0 (0) | 0/0 (0) | 1/1 (11) | 1/1 (8) |
| Psychiatric disorders | 0/0 (0) | 0/0 (0) | 1/1 (11) | 0/0 (0) | 1/1 (8) |
| Sleep disorder | 0/0 (0) | 0/0 (0) | 1/1 (11) | 0/0 (0) | 1/1 (8) |

^*^TEAEs were classified according to MedDRA Version 21.0

^†^FMGX-related TEAEs by SOC and PT for study 1; 1/1 (11%)

% = percentage of the total number of participants per treatment that experienced TEAEs; E = number of times the AEs occurred; FMGX = fosmanogepix; N = number of participants exposed; n = number of participants that experienced TEAEs; IV = intravenous; PBO = placebo; PT = preferred term; SOC = system organ class; TEAEs = treatment emergent adverse events

# **Table S4. FMGX-Related TEAEs Events by SOC and PT for Multiple Doses of IV-infused FMGX (Study 2)**

| **TEAEs by SOC and PT^*^  E/n (%)** | **PBO** | | | **FMGX** | | | | | | **Total**  **(N=51)** |
| --- | --- | --- | --- | --- | --- | --- | --- | --- | --- | --- |
|  | **PBO 1**  **(N=8)** | **PBO 2**  **(N=2)** | **PBO 3**  **(N=2)** | **Cohort A**  **1500/900 mg**  **(N=9)** | **Cohort B**  **900/900 mg (N=6)** | **Cohort C 1000/900 mg**  **(N=6)** | **Cohort D**  **1000/750 mg (N=6)** | **Cohort E**  **1000/850 mg (N=6)** | **Cohort F**  **1000/900 mg (N=6)** |  |
| Any TEAEs | 15/5 (62.5) | 2/1 (50.0) | 6/1 (50.0) | 37/8 (88.9) | 29/6 (100) | 27/5 (83.3) | 34/5 (83.3) | 20/5 (83.3) | 19/6 (100) | 189/42 (82.4) |
| Nervous system disorders | 6/3 (37.5) | 1/1 (50.0) | 2/1 (50.0) | 14/6 (66.7) | 8/6 (100) | 19/5 (83.3) | 15/4 (66.7) | 11/5 (83.3) | 14/6 (100) | 90/37 (72.5) |
| Headache | 1/1 (12.5) | 1/1 (50.0) | 1/1 (50.0) | 10/5 (55.6) | 7/6 (100) | 11/5 (83.3) | 9/4 (66.7) | 8/5 (83.3) | 12/6 (100) | 60/34 (66.7) |
| Dizziness | 2/2 (25.0) | 0/0 (0) | 1/1 (50.0) | 2/2 (22.2) | 1/1 (16.7) | 4/3 (50.0) | 5/3 (50.0) | 1/1 (16.7) | 0/0 (0) | 16/13 (25.5) |
| Somnolence | 2/2 (25.0) | 0/0 (0) | 0/0 (0) | 1/1 (11.1) | 0/0 (0) | 2/2 (33.3) | 0/0 (0) | 2/2 (33.3) | 1/1 (16.7) | 8/8 (15.7) |
| Dizziness Postural | 0/0 (0) | 0/0 (0) | 0/0 (0) | 1/1 (11.1) | 0/0 (0) | 0/0 (0) | 0/0 (0) | 0/0 (0) | 0/0 (0) | 1/1 (2.0) |
| Dysgeusia | 0/0 (0) | 0/0 (0) | 0/0 (0) | 0/0 (0) | 0/0 (0) | 0/0 (0) | 1/1 (16.7) | 0/0 (0) | 0/0 (0) | 1/1 (2.0) |
| Head discomfort | 1/1 (12.5) | 0/0 (0) | 0/0 (0) | 0/0 (0) | 0/0 (0) | 0/0 (0) | 0/0 (0) | 0/0 (0) | 0/0 (0) | 1/1 (2.0) |
| Paresthesia | 0/0 (0) | 0/0 (0) | 0/0 (0) | 0/0 (0) | 0/0 (0) | 2/1 (16.7) | 0/0 (0) | 0/0 (0) | 0/0 (0) | 2/1 (2.0) |
| Syncope | 0/0 (0) | 0/0 (0) | 0/0 (0) | 0/0 (0) | 0/0 (0) | 0/0 (0) | 0/0 (0) | 0/0 (0) | 1/1 (16.7) | 1/1 (2.0) |
| Gastrointestinal disorders | 1/1 (12.5) | 0/0 (0) | 4/1 (50.0) | 17/8 (88.9) | 15/6 (100) | 8/4 (66.7) | 9/5 (83.3) | 3/2 (33.3) | 2/2 (33.3) | 59/29 (56.9) |
| Nausea | 0/0 (0) | 0/0 (0) | 2/1 (50.0) | 9/8 (88.9) | 5/5 (83.3) | 8/4 (66.7) | 5/4 (66.7) | 2/2 (33.3) | 2/2 (33.3) | 33/26 (51.0) |
| Vomiting | 0/0 (0) | 0/0 (0) | 0/0 (0) | 7/3 (33.3) | 7/5 (83.3) | 0/0 (0) | 0/0 (0) | 1/1 (16.7) | 0/0 (0) | 15/9 (17.6) |
| Abdominal pain | 1/1 (12.5) | 0/0 (0) | 2/1 (50.0) | 1/1 (11.1) | 0/0 (0) | 0/0 (0) | 2/2 (33.3) | 0/0 (0) | 0/0 (0) | 6/5 (9.8) |
| Diarrhea | 0/0 (0) | 0/0 (0) | 0/0 (0) | 0/0 (0) | 1/1 (16.7) | 0/0 (0) | 1/1 (16.7) | 0/0 (0) | 0/0 (0) | 2/2 (3.9) |
| Abnormal faeces | 0/0 (0) | 0/0 (0) | 0/0 (0) | 0/0 (0) | 0/0 (0) | 0/0 (0) | 1/1 (16.7) | 0/0 (0) | 0/0 (0) | 1/1 (2.0) |
| Faeces discolored | 0/0 (0) | 0/0 (0) | 0/0 (0) | 0/0 (0) | 1/1 (16.7) | 0/0 (0) | 0/0 (0) | 0/0 (0) | 0/0 (0) | 1/1 (2.0) |
| Flatulence | 0/0 (0) | 0/0 (0) | 0/0 (0) | 0/0 (0) | 1/1 (16.7) | 0/0 (0) | 0/0 (0) | 0/0 (0) | 0/0 (0) | 1/1 (2.0) |
| General disorders and administration site conditions | 4/2 (25.0) | 1/1 (50.0) | 0/0 (0) | 5/3 (33.3) | 5/4 (66.7) | 0/0 (0) | 6/4 (66.7) | 4/2 (33.3) | 1/1 (16.7) | 26/17 (33.3) |
| Chest pain | 1/1 (12.5) | 0/0 (0) | 0/0 (0) | 0/0 (0) | 0/0 (0) | 0/0 (0) | 3/3 (50.0) | 0/0 (0) | 0/0 (0) | 4/4 (7.8) |
| Fatigue | 1/1 (12.5) | 0/0 (0) | 0/0 (0) | 1/1 (11.1) | 1/1 (16.7) | 0/0 (0) | 0/0 (0) | 0/0 (0) | 1/1 (16.7) | 4/4 (7.8) |
| Infusion site irritation | 0/0 (0) | 0/0 (0) | 0/0 (0) | 3/3 (33.3) | 0/0 (0) | 0/0 (0) | 0/0 (0) | 0/0 (0) | 0/0 (0) | 3/3 (5.9) |
| Infusion site pain | 0/0 (0) | 0/0 (0) | 0/0 (0) | 0/0 (0) | 1/1 (16.7) | 0/0 (0) | 1/1 (16.7) | 1/1 (16.7) | 0/0 (0) | 3/3 (5.9) |
| Catheter site inflammation | 0/0 (0) | 0/0 (0) | 0/0 (0) | 1/1 (11.1) | 1/1 (16.7) | 0/0 (0) | 0/0 (0) | 0/0 (0) | 0/0 (0) | 2/2 (3.9) |
| Feeling hot | 0/0 (0) | 0/0 (0) | 0/0 (0) | 0/0 (0) | 0/0 (0) | 0/0 (0) | 2/2 (33.3) | 0/0 (0) | 0/0 (0) | 2/2 (3.9) |
| Infusion site erythema | 1/1 (12.5) | 0/0 (0) | 0/0 (0) | 0/0 (0) | 0/0 (0) | 0/0 (0) | 0/0 (0) | 1/1 (16.7) | 0/0 (0) | 2/2 (3.9) |
| Infusion site induration | 1/1 (12.5) | 0/0 (0) | 0/0 (0) | 0/0 (0) | 0/0 (0) | 0/0 (0) | 0/0 (0) | 1/1 (16.7) | 0/0 (0) | 2/2 (3.9) |
| Infusion site reaction | 0/0 (0) | 1/1 (50.0) | 0/0 (0) | 0/0 (0) | 0/0 (0) | 0/0 (0) | 0/0 (0) | 1/1 (16.7) | 0/0 (0) | 2/2 (3.9) |
| Infusion site inflammation | 0/0 (0) | 0/0 (0) | 0/0 (0) | 0/0 (0) | 1/1 (16.7) | 0/0 (0) | 0/0 (0) | 0/0 (0) | 0/0 (0) | 1/1 (2.0) |
| Injection site inflammation | 0/0 (0) | 0/0 (0) | 0/0 (0) | 0/0 (0) | 1/1 (16.7) | 0/0 (0) | 0/0 (0) | 0/0 (0) | 0/0 (0) | 1/1 (2.0) |
| Musculoskeletal and connective tissue disorders | 0/0 (0) | 0/0 (0) | 0/0 (0) | 1/1 (11.1) | 1/1 (16.7) | 0/0 (0) | 0/0 (0) | 0/0 (0) | 1/1 (16.7) | 3/3 (5.9) |
| Musculoskeletal stiffness | 0/0 (0) | 0/0 (0) | 0/0 (0) | 0/0 (0) | 1/1 (16.7) | 0/0 (0) | 0/0 (0) | 0/0 (0) | 0/0 (0) | 1/1 (2.0) |
| Myalgia | 0/0 (0) | 0/0 (0) | 0/0 (0) | 1/1 (11.1) | 0/0 (0) | 0/0 (0) | 0/0 (0) | 0/0 (0) | 0/0 (0) | 1/1 (2.0) |
| Pain in jaw | 0/0 (0) | 0/0 (0) | 0/0 (0) | 0/0 (0) | 0/0 (0) | 0/0 (0) | 0/0 (0) | 0/0 (0) | 1/1 (16.7) | 1/1 (2.0) |
| Respiratory, thoracic, and mediastinal disorders | 2/1 (12.5) | 0/0 (0) | 0/0 (0) | 0/0 (0) | 0/0 (0) | 0/0 (0) | 1/1 (16.7) | 0/0 (0) | 0/0 (0) | 3/2 (3.9) |
| Dyspnea | 1/1 (12.5) | 0/0 (0) | 0/0 (0) | 0/0 (0) | 0/0 (0) | 0/0 (0) | 1/1 (16.7) | 0/0 (0) | 0/0 (0) | 2/2 (3.9) |
| Epistaxis | 1/1 (12.5) | 0/0 (0) | 0/0 (0) | 0/0 (0) | 0/0 (0) | 0/0 (0) | 0/0 (0) | 0/0 (0) | 0/0 (0) | 1/1 (2.0) |
| Eye disorders | 1/1 (12.5) | 0/0 (0) | 0/0 (0) | 0/0 (0) | 0/0 (0) | 0/0 (0) | 0/0 (0) | 1/1 (16.7) | 0/0 (0) | 2/2 (3.9) |
| Photophobia | 0/0 (0) | 0/0 (0) | 0/0 (0) | 0/0 (0) | 0/0 (0) | 0/0 (0) | 0/0 (0) | 1/1 (16.7) | 0/0 (0) | 1/1 (2.0) |
| Photopsia | 1/1 (12.5) | 0/0 (0) | 0/0 (0) | 0/0 (0) | 0/0 (0) | 0/0 (0) | 0/0 (0) | 0/0 (0) | 0/0 (0) | 1/1 (2.0) |
| Vascular disorders | 0/0 (0) | 0/0 (0) | 0/0 (0) | 0/0 (0) | 0/0 (0) | 0/0 (0) | 1/1 (16.7) | 1/1 (16.7) | 0/0 (0) | 2/2 (3.9) |
| Flushing | 0/0 (0) | 0/0 (0) | 0/0 (0) | 0/0 (0) | 0/0 (0) | 0/0 (0) | 0/0 (0) | 1/1 (16.7) | 0/0 (0) | 1/1 (2.0) |
| Pallor | 0/0 (0) | 0/0 (0) | 0/0 (0) | 0/0 (0) | 0/0 (0) | 0/0 (0) | 1/1 (16.7) | 0/0 (0) | 0/0 (0) | 1/1 (2.0) |
| Injury, poisoning, and procedural complications | 0/0 (0) | 0/0 (0) | 0/0 (0) | 0/0 (0) | 0/0 (0) | 0/0 (0) | 0/0 (0) | 0/0 (0) | 1/1 (16.7) | 1/1 (2.0) |
| Wound | 0/0 (0) | 0/0 (0) | 0/0 (0) | 0/0 (0) | 0/0 (0) | 0/0 (0) | 0/0 (0) | 0/0 (0) | 1/1 (16.7) | 1/1 (2.0) |
| Metabolism and nutrition disorders | 0/0 (0) | 0/0 (0) | 0/0 (0) | 0/0 (0) | 0/0 (0) | 0/0 (0) | 1/1 (16.7) | 0/0 (0) | 0/0 (0) | 1/1 (2.0) |
| Decreased appetite | 0/0 (0) | 0/0 (0) | 0/0 (0) | 0/0 (0) | 0/0 (0) | 0/0 (0) | 1/1 (16.7) | 0/0 (0) | 0/0 (0) | 1/1 (2.0) |
| Psychiatric disorders | 0/0 (0) | 0/0 (0) | 0/0 (0) | 0/0 (0) | 0/0 (0) | 0/0 (0) | 1/1 (16.7) | 0/0 (0) | 0/0 (0) | 1/1 (2.0) |
| Emotional disorder | 0/0 (0) | 0/0 (0) | 0/0 (0) | 0/0 (0) | 0/0 (0) | 0/0 (0) | 1/1 (16.7) | 0/0 (0) | 0/0 (0) | 1/1 (2.0) |
| Skin and subcutaneous tissue disorders | 1/1 (12.5) | 0/0 (0) | 0/0 (0) | 0/0 (0) | 0/0 (0) | 0/0 (0) | 0/0 (0) | 0/0 (0) | 0/0 (0) | 1/1 (2.0) |
| Rash | 1/1 (12.5) | 0/0 (0) | 0/0 (0) | 0/0 (0) | 0/0 (0) | 0/0 (0) | 0/0 (0) | 0/0 (0) | 0/0 (0) | 1/1 (2.0) |

^*^TEAEs were classified according to MedDRA Version 21.0

Cohorts: PBO 1 = Placebo BID on Day 1 and QD on Days 2 to 7 IV infusion; PBO 2 = Placebo BID on Days 1 and 2 and QD on Days 3 to 7 IV infusion; PBO 3 = Placebo BID on Day 1 and QD on Days 2 to 7 IV infusion with ondansetron; A = 1500 mg BID on Day 1 and 900 mg QD on Days 2 to 7 FMGX IV infusion; B = 900 mg BID on Days 1 and 2 and 900 mg QD on Days 3 to 7 FMGX IV infusion; C = 1000 mg BID on Day 1 and 900 mg QD on Days 2 to 7 FMGX IV infusion with ondansetron; D = 1000 mg BID on Day 1 and 750 mg QD on Days 2 to 7 FMGX IV infusion; E = 1000 mg BID on Day 1 and 850 mg QD on Days 2 to 7 FMGX IV infusion; F = 1000 mg BID on Day 1 and 900 mg QD on Days 2 to 7 FMGX IV infusion

% = percentage of the total number of participants per treatment that experienced TEAEs; BID, twice daily; E = number of times the TEAEs occurred; FMGX = fosmanogepix; IV = intravenous; N = number of participants exposed; n = number of participants that experienced TEAEs; PBO = placebo; PT = preferred term; QD, once daily; SOC = system organ class; TEAEs = treatment-emergent adverse events

# **Table S5. All TEAEs by SOC and PT for Multiple Doses of IV-infused FMGX and Summary of Moderate and Severe TEAEs**

| **TEAEs by SOC and PT^*^  E/n (%)** | **PBO** | | | **FMGX** | | | | | | **Total**  **(N=51)** |
| --- | --- | --- | --- | --- | --- | --- | --- | --- | --- | --- |
|  | **PBO 1**  **(N=8)** | **PBO 2**  **(N=2)** | **PBO 3**  **(N=2)** | **Cohort A**  **1500/900 mg**  **(N=9)** | **Cohort B**  **900/900 mg (N=6)** | **Cohort C 1000/900 mg**  **(N=6)** | **Cohort D**  **1000/750 mg (N=6)** | **Cohort E**  **1000/850 mg (N=6)** | **Cohort F**  **1000/900 mg (N=6)** |  |
| Any AEs | 40/8 (100) | 7/2 (100) | 23/2 (100) | 67/9 (100) | 41/6 (100) | 51/6 (100) | 47/6 (100) | 40/6 (100) | 41/6 (100) | 357/51 (100) |
| **Nervous system disorders** | 12/6 (75.0) | 2/2 (100) | 5/2 (100) | 20/9 (100) | 10/6 (100) | 26/6 (100) | 16/5 (83.3) | 14/5 (83.3) | 16/6 (100) | 121/47 (92.2) |
| Headache | 2/2 (25.0) | 2/2 (100) | 2/1 (50.0) | 12/7 (77.8) | 8/6 (100) | 13/6 (100) | 10/5 (83.3) | 9/5 (83.3) | 12/6 (100) | 70/40 (78.4) |
| Moderate | 0/0 (0.0) | 0/0 (0.0) | 0/0 (0.0) | 2/2 (22.2) | 5/5 (83.3) | 1/1 (16.7) | 1/1 (16.7) | 1/1 (16.7) | 1/1 (16.7) | 11/11 (21.6) |
| Dizziness | 3/3 (37.5) | 0/0 (0.0) | 1/1 (50.0) | 3/3 (33.3) | 2/1 (16.7) | 6/5 (83.3) | 5/3 (50.0) | 2/2 (33.3) | 0/0 (0.0) | 22/18 (35.3) |
| Moderate | 0/0 (0.0) | 0/0 (0.0) | 0/0 (0.0) | 1/1 (11.1) | 0/0 (0.0) | 0/0 (0.0) | 0/0 (0.0) | 0/0 (0.0) | 0/0 (0.0) | 1/1 (2.0) |
| Somnolence | 2/2 (25.0) | 0/0 (0.0) | 1/1 (50.0) | 1/1 (11.1) | 0/0 (0.0) | 4/3 (50.0) | 0/0 (0.0) | 2/2 (33.3) | 2/1 (16.7) | 12/10 (19.6) |
| Dysgeusia | 1/1 (12.5) | 0/0 (0.0) | 1/1 (50.0) | 1 1 (11.1) | 0/0 (0.0) | 0/0 (0.0) | 1/1 (16.7) | 0/0 (0.0) | 0/0 (0.0) | 4/4 (7.8) |
| Dizziness postural | 1/1 (12.5) | 0/0 (0.0) | 0/0 (0.0) | 1/1 (11.1) | 0/0 (0.0) | 0/0 (0.0) | 0/0 (0.0) | 0/0 (0.0) | 0/0 (0.0) | 2/2 (3.9) |
| Head discomfort | 1/1 (12.5) | 0/0 (0.0) | 0/0 (0.0) | 1/1 (11.1) | 0/0 (0.0) | 0/0 (0.0) | 0/0 (0.0) | 0/0 (0.0) | 0/0 (0.0) | 2/2 (3.9) |
| Presyncope | 0/0 (0.0) | 0/0 (0.0) | 0/0 (0.0) | 0/0 (0.0) | 0/0 (0.0) | 0/0 (0.0) | 0/0 (0.0) | 1/1 (16.7) | 1/1 (16.7) | 2/2 (3.9) |
| Disturbance in attention | 0/0 (0.0) | 0/0 (0.0) | 0/0 (0.0) | 1/1 (11.1) | 0/0 (0.0) | 0/0 (0.0) | 0/0 (0.0) | 0/0 (0.0) | 0/0 (0.0) | 1/1 (2.0) |
| Hypoesthesia | 2/1 (12.5) | 0/0 (0.0) | 0/0 (0.0) | 0/0 (0.0) | 0/0 (0.0) | 0/0 (0.0) | 0/0 (0.0) | 0/0 (0.0) | 0/0 (0.0) | 2/1 (2.0) |
| Paresthesia | 0/0 (0.0) | 0/0 (0.0) | 0/0 (0.0) | 0/0 (0.0) | 0/0 (0.0) | 3/1 (16.7) | 0/0 (0.0) | 0/0 (0.0) | 0/0 (0.0) | 3/1 (2.0) |
| Syncope | 0/0 (0.0) | 0/0 (0.0) | 0/0 (0.0) | 0/0 (0.0) | 0/0 (0.0) | 0/0 (0.0) | 0/0 (0.0) | 0/0 (0.0) | 1/1 (16.7) | 1/1 (2.0) |
| Severe | 0/0 (0.0) | 0/0 (0.0) | 0/0 (0.0) | 0/0 (0.0) | 0/0 (0.0) | 0/0 (0.0) | 0/0 (0.0) | 0/0 (0.0) | 1/1 (16.7) | 1/1 (2.0) |
| **General disorders and administration site conditions** | 16/6 (75.0) | 2/1 (50.0) | 4/2 (100) | 10/6 (66.7) | 11/5 (83.3) | 9/5 (83.3) | 12/6 (100) | 12/5 (83.3) | 17/6 (100) | 93/42 (82.4) |
| Catheter site pain | 5/5 (62.5) | 0/0 (0.0) | 3/2 (100) | 1/1 (11.1) | 1/1 (16.7) | 3/3 (50.0) | 5/5 (83.3) | 2/2 (33.3) | 4/4 (66.7) | 24/23 (45.1) |
| Fatigue | 2/2 (25.0) | 0/0 (0.0) | 1/1 (50.0) | 1/1 (11.1) | 2/2 (33.3) | 1/1 (16.7) | 0/0 (0.0) | 0/0 (0.0) | 1/1 (16.7) | 8/8 (15.7) |
| Infusion site irritation | 0/0 (0.0) | 0/0 (0.0) | 0/0 (0.0) | 4/4 (44.4) | 2/1 (16.7) | 0/0 (0.0) | 0/0 (0.0) | 0/0 (0.0) | 1/1 (16.7) | 7/6 (11.8) |
| Moderate | 0/0 (0.0) | 0/0 (0.0) | 0/0 (0.0) | 2/2 (22.2) | 0/0 (0.0) | 0/0 (0.0) | 0/0 (0.0) | 0/0 (0.0) | 1/1 (16.7) | 3/3 (5.9) |
| Infusion site pain | 1/1 (12.5) | 0/0 (0.0) | 0/0 (0.0) | 0/0 (0.0) | 1/1 (16.7) | 0/0 (0.0) | 1/1 (16.7) | 2/2 (33.3) | 1/1 (16.7) | 6/6 (11.8) |
| Moderate | 0/0 (0.0) | 0/0 (0.0) | 0/0 (0.0) | 0/0 (0.0) | 0/0 (0.0) | 0/0 (0.0) | 1/1 (16.7) | 0/0 (0.0) | 0/0 (0.0) | 1/1 (2.0) |
| Asthenia | 1/1 (12.5) | 0/0 (0.0) | 0/0 (0.0) | 1/1 (11.1) | 1/1 (16.7) | 1/1 (16.7) | 0/0 (0.0) | 1/1 (16.7) | 0/0 (0.0) | 5/5 (9.8) |
| Catheter site erythema | 0/0 (0.0) | 0/0 (0.0) | 0/0 (0.0) | 0/0 (0.0) | 0/0 (0.0) | 2/2 (33.3) | 1/1 (16.7) | 1/1 (16.7) | 1/1 (16.7) | 5/5 (9.8) |
| Catheter site hematoma | 2/2 (25.0) | 0/0 (0.0) | 0/0 (0.0) | 0/0 (0.0) | 0/0 (0.0) | 1/1 (16.7) | 0/0 (0.0) | 1/1 (16.7) | 1/1 (16.7) | 5/5 (9.8) |
| Catheter site swelling | 0/0 (0.0) | 0/0 (0.0) | 0/0 (0.0) | 1/1 (11.1) | 0/0 (0.0) | 0/0 (0.0) | 0/0 (0.0) | 0/0 (0.0) | 5/4 (66.7) | 6/5 (9.8) |
| Moderate | 0/0 (0.0) | 0/0 (0.0) | 0/0 (0.0) | 1/1 (11.1) | 0/0 (0.0) | 0/0 (0.0) | 0/0 (0.0) | 0/0 (0.0) | 0/0 (0.0) | 1/1 (2.0) |
| Catheter site inflammation | 0/0 (0.0) | 0/0 (0.0) | 0/0 (0.0) | 2/2 (22.2) | 2/2 (33.3) | 0/0 (0.0) | 0/0 (0.0) | 0/0 (0.0) | 0/0 (0.0) | 4/4 (7.8) |
| Chest pain | 1/1 (12.5) | 0/0 (0.0) | 0/0 (0.0) | 0/0 (0.0) | 0/0 (0.0) | 0/0 (0.0) | 3/3 (50.0) | 0/0 (0.0) | 0/0 (0.0) | 4/4 (7.8) |
| Infusion site induration | 2/2 (25.0) | 0/0 (0.0) | 0/0 (0.0) | 0/0 (0.0) | 0/0 (0.0) | 0/0 (0.0) | 0/0 (0.0) | 1/1 (16.7) | 0/0 (0.0) | 3/3 (5.9) |
| Feeling hot | 0/0 (0.0) | 0/0 (0.0) | 0/0 (0.0) | 0/0 (0.0) | 0/0 (0.0) | 0/0 (0.0) | 2/2 (33.3) | 0/0 (0.0) | 0/0 (0.0) | 2/2 (3.9) |
| Infusion site erythema | 1/1 (12.5) | 0/0 (0.0) | 0/0 (0.0) | 0/0 (0.0) | 0/0 (0.0) | 0/0 (0.0) | 0/0 (0.0) | 1/1 (16.7) | 0/0 (0.0) | 2/2 (3.9) |
| Infusion site inflammation | 0/0 (0.0) | 0/0 (0.0) | 0/0 (0.0) | 0/0 (0.0) | 1/1 (16.7) | 0/0 (0.0) | 0/0 (0.0) | 0/0 (0.0) | 1/1 (16.7) | 2/2 (3.9) |
| Moderate | 0/0 (0.0) | 0/0 (0.0) | 0/0 (0.0) | 0/0 (0.0) | 1/1 (16.7) | 0/0 (0.0) | 0/0 (0.0) | 0/0 (0.0) | 0/0 (0.0) | 1/1 (2.0) |
| Infusion site reaction | 0/0 (0.0) | 1/1 (50.0) | 0/0 (0.0) | 0/0 (0.0) | 0/0 (0.0) | 0/0 (0.0) | 0/0 (0.0) | 1/1 (16.7) | 0/0 (0.0) | 2/2 (3.9) |
| Infusion site swelling | 0/0 (0.0) | 0/0 (0.0) | 0/0 (0.0) | 0/0 (0.0) | 0/0 (0.0) | 0/0 (0.0) | 0/0 (0.0) | 0/0 (0.0) | 2/2 (33.3) | 2/2 (3.9) |
| Hunger | 1/1 (12.5) | 0/0 (0.0) | 0/0 (0.0) | 0/0 (0.0) | 0/0 (0.0) | 0/0 (0.0) | 0/0 (0.0) | 0/0 (0.0) | 0/0 (0.0) | 1/1 (2.0) |
| Influenza like illness | 0/0 (0.0) | 1/1 (50.0) | 0/0 (0.0) | 0/0 (0.0) | 0/0 (0.0) | 0/0 (0.0) | 0/0 (0.0) | 0/0 (0.0) | 0/0 (0.0) | 1/1 (2.0) |
| Infusion site extravasation | 0/0 (0.0) | 0/0 (0.0) | 0/0 (0.0) | 0/0 (0.0) | 0/0 (0.0) | 0/0 (0.0) | 0/0 (0.0) | 1/1 (16.7) | 0/0 (0.0) | 1/1 (2.0) |
| Moderate | 0/0 (0.0) | 0/0 (0.0) | 0/0 (0.0) | 0/0 (0.0) | 0/0 (0.0) | 0/0 (0.0) | 0/0 (0.0) | 1/1 (16.7) | 0/0 (0.0) | 1/1 (2.0) |
| Injection site inflammation | 0/0 (0.0) | 0/0 (0.0) | 0/0 (0.0) | 0/0 (0.0) | 1/1 (16.7) | 0/0 (0.0) | 0/0 (0.0) | 0/0 (0.0) | 0/0 (0.0) | 1/1 (2.0) |
| Moderate | 0/0 (0.0) | 0/0 (0.0) | 0/0 (0.0) | 0/0 (0.0) | 1/1 (16.7) | 0/0 (0.0) | 0/0 (0.0) | 0/0 (0.0) | 0/0 (0.0) | 1/1 (2.0) |
| Injection site pain | 0/0 (0.0) | 0/0 (0.0) | 0/0 (0.0) | 0/0 (0.0) | 0/0 (0.0) | 1/1 (16.7) | 0/0 (0.0) | 0/0 (0.0) | 0/0 (0.0) | 1/1 (2.0) |
| Oedema | 0/0 (0.0) | 0/0 (0.0) | 0/0 (0.0) | 0/0 (0.0) | 0/0 (0.0) | 0/0 (0.0) | 0/0 (0.0) | 1/1 (16.7) | 0/0 (0.0) | 1/1 (2.0) |
| Moderate | 0/0 (0.0) | 0/0 (0.0) | 0/0 (0.0) | 0/0 (0.0) | 0/0 (0.0) | 0/0 (0.0) | 0/0 (0.0) | 1/1 (16.7) | 0/0 (0.0) | 1/1 (2.0) |
| **Gastrointestinal disorders** | 3/2 (25.0) | 1/1 (50.0) | 13/2 (100) | 19/9 (100) | 15/6 (100) | 13/5 (83.3) | 12/6 (100) | 8/4 (66.7) | 3/3 (50.0) | 87/38 (74.5) |
| Nausea | 0/0 (0.0) | 0/0 (0.0) | 2/1 (50.0) | 10/9 (100) | 5/5 (83.3) | 8/4 (66.7) | 6/4 (66.7) | 2/2 (33.3) | 2/2 (33.3) | 35/27 (52.9) |
| Moderate | 0/0 (0.0) | 0/0 (0.0) | 0/0 (0.0) | 3/3 (33.3) | 4/4 (66.7) | 0/0 (0.0) | 1/1 (16.7) | 0/0 (0.0) | 1/1 (16.7) | 9/9 (17.6) |
| Abdominal pain | 2/2 (25.0) | 0/0 (0.0) | 6/2 (100) | 1/1 (11.1) | 0/0 (0.0) | 3/2 (33.3) | 2/2 (33.3) | 0/0 (0.0) | 1/1 (16.7) | 15/10 (19.6) |
| Vomiting | 0/0 (0.0) | 0/0 (0.0) | 0/0 (0.0) | 7/3 (33.3) | 7/5 (83.3) | 0/0 (0.0) | 1/1 (16.7) | 1/1 (16.7) | 0/0 (0.0) | 16/10 (19.6) |
| Moderate | 0/0 (0.0) | 0/0 (0.0) | 0/0 (0.0) | 4/3 (33.3) | 7/5 (83.3) | 0/0 (0.0) | 1/1 (16.7) | 0/0 (0.0) | 0/0 (0.0) | 12/9 (17.6) |
| Diarrhea | 0/0 (0.0) | 1/1 (50.0) | 0/0 (0.0) | 0/0 (0.0) | 1/1 (16.7) | 0/0 (0.0) | 1/1 (16.7) | 2/2 (33.3) | 0/0 (0.0) | 5/5 (9.8) |
| Constipation | 0/0 (0.0) | 0/0 (0.0) | 2/2 (100) | 0/0 (0.0) | 0/0 (0.0) | 2/1 (16.7) | 0/0 (0.0) | 0/0 (0.0) | 0/0 (0.0) | 4/3 (5.9) |
| Dyspepsia | 1/1 (12.5) | 0/0 (0.0) | 0/0 (0.0) | 1/1 (11.1) | 0/0 (0.0) | 0/0 (0.0) | 0/0 (0.0) | 1/1 (16.7) | 0/0 (0.0) | 3/3 (5.9) |
| Moderate | 0/0 (0.0) | 0/0 (0.0) | 0/0 (0.0) | 1/1 (11.1) | 0/0 (0.0) | 0/0 (0.0) | 0/0 (0.0) | 0/0 (0.0) | 0/0 (0.0) | 1/1 (2.0) |
| Abdominal discomfort | 0/0 (0.0) | 0/0 (0.0) | 0/0 (0.0) | 0/0 (0.0) | 0/0 (0.0) | 0/0 (0.0) | 1/1 (16.7) | 0/0 (0.0) | 0/0 (0.0) | 1/1 (2.0) |
| Abnormal faces | 0/0 (0.0) | 0/0 (0.0) | 0/0 (0.0) | 0/0 (0.0) | 0/0 (0.0) | 0/0 (0.0) | 1/1 (16.7) | 0/0 (0.0) | 0/0 (0.0) | 1/1 (2.0) |
| Dry mouth | 0/0 (0.0) | 0/0 (0.0) | 0/0 (0.0) | 0/0 (0.0) | 0/0 (0.0) | 0/0 (0.0) | 0/0 (0.0) | 1/1 (16.7) | 0/0 (0.0) | 1/1 (2.0) |
| Faces discolored | 0/0 (0.0) | 0/0 (0.0) | 0/0 (0.0) | 0/0 (0.0) | 1/1 (16.7) | 0/0 (0.0) | 0/0 (0.0) | 0/0 (0.0) | 0/0 (0.0) | 1/1 (2.0) |
| Faces hard | 0/0 (0.0) | 0/0 (0.0) | 1/1 (50.0) | 0/0 (0.0) | 0/0 (0.0) | 0/0 (0.0) | 0/0 (0.0) | 0/0 (0.0) | 0/0 (0.0) | 1/1 (2.0) |
| Flatulence | 0/0 (0.0) | 0/0 (0.0) | 0/0 (0.0) | 0/0 (0.0) | 1/1 (16.7) | 0/0 (0.0) | 0/0 (0.0) | 0/0 (0.0) | 0/0 (0.0) | 1/1 (2.0) |
| Gastrointestinal sounds abnormal | 0/0 (0.0) | 0/0 (0.0) | 1/1 (50.0) | 0/0 (0.0) | 0/0 (0.0) | 0/0 (0.0) | 0/0 (0.0) | 0/0 (0.0) | 0/0 (0.0) | 1/1 (2.0) |
| Lip dry | 0/0 (0.0) | 0/0 (0.0) | 0/0 (0.0) | 0/0 (0.0) | 0/0 (0.0) | 0/0 (0.0) | 0/0 (0.0) | 1/1 (16.7) | 0/0 (0.0) | 1/1 (2.0) |
| Paresthesia oral | 0/0 (0.0) | 0/0 (0.0) | 1/1 (50.0) | 0/0 (0.0) | 0/0 (0.0) | 0/0 (0.0) | 0/0 (0.0) | 0/0 (0.0) | 0/0 (0.0) | 1/1 (2.0) |
| **Musculoskeletal and connective tissue disorders** | 1/1 (12.5) | 1/1 (50.0) | 1/1 (50.0) | 5/4 (44.4) | 3/3 (50.0) | 0/0 (0.0) | 0/0 (0.0) | 1/1 (16.7) | 2/1 (16.7) | 14/12 (23.5) |
| Back pain | 1/1 (12.5) | 1/1 (50.0) | 0/0 (0.0) | 0/0 (0.0) | 2/2 (33.3) | 0/0 (0.0) | 0/0 (0.0) | 1/1 (16.7) | 1/1 (16.7) | 6/6 (11.8) |
| Moderate | 0/0 (0.0) | 1/1 (50.0) | 0/0 (0.0) | 0/0 (0.0) | 1/1 (16.7) | 0/0 (0.0) | 0/0 (0.0) | 0/0 (0.0) | 0/0 (0.0) | 2/2 (3.9) |
| Muscle contracture | 0/0 (0.0) | 0/0 (0.0) | 0/0 (0.0) | 1/1 (11.1) | 0/0 (0.0) | 0/0 (0.0) | 0/0 (0.0) | 0/0 (0.0) | 0/0 (0.0) | 1/1 (2.0) |
| Moderate | 0/0 (0.0) | 0/0 (0.0) | 0/0 (0.0) | 1/1 (11.1) | 0/0 (0.0) | 0/0 (0.0) | 0/0 (0.0) | 0/0 (0.0) | 0/0 (0.0) | 1/1 (2.0) |
| Muscle twitching | 0/0 (0.0) | 0/0 (0.0) | 0/0 (0.0) | 1/1 (11.1) | 0/0 (0.0) | 0/0 (0.0) | 0/0 (0.0) | 0/0 (0.0) | 0/0 (0.0) | 1/1 (2.0) |
| Muscular weakness | 0/0 (0.0) | 0/0 (0.0) | 0/0 (0.0) | 1 1 (11.1) | 0/0 (0.0) | 0/0 (0.0) | 0/0 (0.0) | 0/0 (0.0) | 0/0 (0.0) | 1 1 (2.0) |
| Musculoskeletal stiffness | 0/0 (0.0) | 0/0 (0.0) | 0/0 (0.0) | 0/0 (0.0) | 1/1 (16.7) | 0/0 (0.0) | 0/0 (0.0) | 0/0 (0.0) | 0/0 (0.0) | 1/1 (2.0) |
| Myalgia | 0/0 (0.0) | 0/0 (0.0) | 0/0 (0.0) | 1/1 (11.1) | 0/0 (0.0) | 0/0 (0.0) | 0/0 (0.0) | 0/0 (0.0) | 0/0 (0.0) | 1/1 (2.0) |
| Neck pain | 0/0 (0.0) | 0/0 (0.0) | 1/1 (50.0) | 0/0 (0.0) | 0/0 (0.0) | 0/0 (0.0) | 0/0 (0.0) | 0/0 (0.0) | 0/0 (0.0) | 1/1 (2.0) |
| Pain in extremity | 0/0 (0.0) | 0/0 (0.0) | 0/0 (0.0) | 1/1 (11.1) | 0/0 (0.0) | 0/0 (0.0) | 0/0 (0.0) | 0/0 (0.0) | 0/0 (0.0) | 1/1 (2.0) |
| Pain in jaw | 0/0 (0.0) | 0/0 (0.0) | 0/0 (0.0) | 0/0 (0.0) | 0/0 (0.0) | 0/0 (0.0) | 0/0 (0.0) | 0/0 (0.0) | 1/1 (16.7) | 1/1 (2.0) |
| **Respiratory, thoracic, and mediastinal disorders** | 2/1 (12.5) | 0/0 (0.0) | 0/0 (0.0) | 2/1 (11.1) | 0/0 (0.0) | 1/1 (16.7) | 2/2 (33.3) | 1/1 (16.7) | 0/0 (0.0) | 8/6 (11.8) |
| Dyspnea | 1/1 (12.5) | 0/0 (0.0) | 0/0 (0.0) | 0/0 (0.0) | 0/0 (0.0) | 0/0 (0.0) | 1/1 (16.7) | 1/1 (16.7) | 0/0 (0.0) | 3/3 (5.9) |
| Epistaxis | 1/1 (12.5) | 0/0 (0.0) | 0/0 (0.0) | 1/1 (11.1) | 0/0 (0.0) | 0/0 (0.0) | 0/0 (0.0) | 0/0 (0.0) | 0/0 (0.0) | 2/2 (3.9) |
| Nasal congestion | 0/0 (0.0) | 0/0 (0.0) | 0/0 (0.0) | 1/1 (11.1) | 0/0 (0.0) | 1/1 (16.7) | 0/0 (0.0) | 0/0 (0.0) | 0/0 (0.0) | 2/2 (3.9) |
| Hyperventilation | 0/0 (0.0) | 0/0 (0.0) | 0/0 (0.0) | 0/0 (0.0) | 0/0 (0.0) | 0/0 (0.0) | 1/1 (16.7) | 0/0 (0.0) | 0/0 (0.0) | 1/1 (2.0) |
| **Psychiatric disorders** | 0/0 (0.0) | 0/0 (0.0) | 0/0 (0.0) | 2/2 (22.2) | 0/0 (0.0) | 1/1 (16.7) | 1/1 (16.7) | 2/2 (33.3) | 0/0 (0.0) | 6/6 (11.8) |
| Emotional disorder | 0/0 (0.0) | 0/0 (0.0) | 0/0 (0.0) | 0/0 (0.0) | 0/0 (0.0) | 0/0 (0.0) | 1/1 (16.7) | 1/1 (16.7) | 0/0 (0.0) | 2/2 (3.9) |
| Agitation | 0/0 (0.0) | 0/0 (0.0) | 0/0 (0.0) | 1/1 (11.1) | 0/0 (0.0) | 0/0 (0.0) | 0/0 (0.0) | 0/0 (0.0) | 0/0 (0.0) | 1/1 (2.0) |
| Nightmare | 0/0 (0.0) | 0/0 (0.0) | 0/0 (0.0) | 0/0 (0.0) | 0/0 (0.0) | 0/0 (0.0) | 0/0 (0.0) | 1/1 (16.7) | 0/0 (0.0) | 1/1 (2.0) |
| Panic attack | 0/0 (0.0) | 0/0 (0.0) | 0/0 (0.0) | 0/0 (0.0) | 0/0 (0.0) | 1/1 (16.7) | 0/0 (0.0) | 0/0 (0.0) | 0/0 (0.0) | 1/1 (2.0) |
| Psychogenic tremor | 0/0 (0.0) | 0/0 (0.0) | 0/0 (0.0) | 1/1 (11.1) | 0/0 (0.0) | 0/0 (0.0) | 0/0 (0.0) | 0/0 (0.0) | 0/0 (0.0) | 1/1 (2.0) |
| **Vascular disorders** | 0/0 (0.0) | 0/0 (0.0) | 0/0 (0.0) | 1/1 (11.1) | 1/1 (16.7) | 1/1 (16.7) | 2/2 (33.3) | 1/1 (16.7) | 0/0 (0.0) | 6/6 (11.8) |
| Hematoma | 0/0 (0.0) | 0/0 (0.0) | 0/0 (0.0) | 1/1 (11.1) | 0/0 (0.0) | 0/0 (0.0) | 1/1 (16.7) | 0/0 (0.0) | 0/0 (0.0) | 2/2 (3.9) |
| Flushing | 0/0 (0.0) | 0/0 (0.0) | 0/0 (0.0) | 0/0 (0.0) | 0/0 (0.0) | 0/0 (0.0) | 0/0 (0.0) | 1/1 (16.7) | 0/0 (0.0) | 1/1 (2.0) |
| Pallor | 0/0 (0.0) | 0/0 (0.0) | 0/0 (0.0) | 0/0 (0.0) | 0/0 (0.0) | 0/0 (0.0) | 1/1 (16.7) | 0/0 (0.0) | 0/0 (0.0) | 1/1 (2.0) |
| Phlebitis | 0/0 (0.0) | 0/0 (0.0) | 0/0 (0.0) | 0/0 (0.0) | 0/0 (0.0) | 1/1 (16.7) | 0/0 (0.0) | 0/0 (0.0) | 0/0 (0.0) | 1/1 (2.0) |
| Venous occlusion | 0/0 (0.0) | 0/0 (0.0) | 0/0 (0.0) | 0/0 (0.0) | 1/1 (16.7) | 0/0 (0.0) | 0/0 (0.0) | 0/0 (0.0) | 0/0 (0.0) | 1/1 (2.0) |
| **Skin and subcutaneous tissue disorders** | 2/2 (25.0) | 0/0 (0.0) | 0/0 (0.0) | 2/2 (22.2) | 0/0 (0.0) | 0/0 (0.0) | 0/0 (0.0) | 0/0 (0.0) | 1/1 (16.7) | 5/5 (9.8) |
| Alopecia | 1/1 (12.5) | 0/0 (0.0) | 0/0 (0.0) | 1/1 (11.1) | 0/0 (0.0) | 0/0 (0.0) | 0/0 (0.0) | 0/0 (0.0) | 0/0 (0.0) | 2/2 (3.9) |
| Rash | 1/1 (12.5) | 0/0 (0.0) | 0/0 (0.0) | 0/0 (0.0) | 0/0 (0.0) | 0/0 (0.0) | 0/0 (0.0) | 0/0 (0.0) | 1/1 (16.7) | 2/2 (3.9) |
| Skin disorder | 0/0 (0.0) | 0/0 (0.0) | 0/0 (0.0) | 1/1 (11.1) | 0/0 (0.0) | 0/0 (0.0) | 0/0 (0.0) | 0/0 (0.0) | 0/0 (0.0) | 1/1 (2.0) |
| Eye disorders | 1/1 (12.5) | 0/0 (0.0) | 0/0 (0.0) | 2/2 (22.2) | 0/0 (0.0) | 0/0 (0.0) | 0/0 (0.0) | 1/1 (16.7) | 0/0 (0.0) | 4/4 (7.8) |
| Photophobia | 0/0 (0.0) | 0/0 (0.0) | 0/0 (0.0) | 1/1 (11.1) | 0/0 (0.0) | 0/0 (0.0) | 0/0 (0.0) | 1/1 (16.7) | 0/0 (0.0) | 2/2 (3.9) |
| Eye pain | 0/0 (0.0) | 0/0 (0.0) | 0/0 (0.0) | 1/1 (11.1) | 0/0 (0.0) | 0/0 (0.0) | 0/0 (0.0) | 0/0 (0.0) | 0/0 (0.0) | 1/1 (2.0) |
| Photopsia | 1/1 (12.5) | 0/0 (0.0) | 0/0 (0.0) | 0/0 (0.0) | 0/0 (0.0) | 0/0 (0.0) | 0/0 (0.0) | 0/0 (0.0) | 0/0 (0.0) | 1/1 (2.0) |
| **Infections and infestations** | 0/0 (0.0) | 0/0 (0.0) | 0/0 (0.0) | 2/2 (22.2) | 1/1 (16.7) | 0/0 (0.0) | 1/1 (16.7) | 0/0 (0.0) | 0/0 (0.0) | 4/4 (7.8) |
| Nasopharyngitis | 0/0 (0.0) | 0/0 (0.0) | 0/0 (0.0) | 1/1 (11.1) | 0/0 (0.0) | 0/0 (0.0) | 1/1 (16.7) | 0/0 (0.0) | 0/0 (0.0) | 2/2 (3.9) |
| Conjunctivitis | 0/0 (0.0) | 0/0 (0.0) | 0/0 (0.0) | 1/1 (11.1) | 0/0 (0.0) | 0/0 (0.0) | 0/0 (0.0) | 0/0 (0.0) | 0/0 (0.0) | 1/1 (2.0) |
| Urinary tract infection | 0/0 (0.0) | 0/0 (0.0) | 0/0 (0.0) | 0/0 (0.0) | 1/1 (16.7) | 0/0 (0.0) | 0/0 (0.0) | 0/0 (0.0) | 0/0 (0.0) | 1/1 (2.0) |
| Moderate | 0/0 (0.0) | 0/0 (0.0) | 0/0 (0.0) | 0/0 (0.0) | 1/1 (16.7) | 0/0 (0.0) | 0/0 (0.0) | 0/0 (0.0) | 0/0 (0.0) | 1/1 (2.0) |
| **Metabolism and nutrition disorders** | 0/0 (0.0) | 0/0 (0.0) | 0/0 (0.0) | 2/2 (22.2) | 0/0 (0.0) | 0/0 (0.0) | 1/1 (16.7) | 0/0 (0.0) | 0/0 (0.0) | 3/3 (5.9) |
| Decreased appetite | 0/0 (0.0) | 0/0 (0.0) | 0/0 (0.0) | 1/1 (11.1) | 0/0 (0.0) | 0/0 (0.0) | 1/1 (16.7) | 0/0 (0.0) | 0/0 (0.0) | 2/2 (3.9) |
| Increased appetite | 0/0 (0.0) | 0/0 (0.0) | 0/0 (0.0) | 1/1 (11.1) | 0/0 (0.0) | 0/0 (0.0) | 0/0 (0.0) | 0/0 (0.0) | 0/0 (0.0) | 1/1 (2.0) |
| **Injury, poisoning, and procedural complications** | 1/1 (12.5) | 0/0 (0.0) | 0/0 (0.0) | 0/0 (0.0) | 0/0 (0.0) | 0/0 (0.0) | 0/0 (0.0) | 0/0 (0.0) | 1/1 (16.7) | 2/2 (3.9) |
| Procedural dizziness | 1/1 (12.5) | 0/0 (0.0) | 0/0 (0.0) | 0/0 (0.0) | 0/0 (0.0) | 0/0 (0.0) | 0/0 (0.0) | 0/0 (0.0) | 0/0 (0.0) | 1/1 (2.0) |
| Wound | 0/0 (0.0) | 0/0 (0.0) | 0/0 (0.0) | 0/0 (0.0) | 0/0 (0.0) | 0/0 (0.0) | 0/0 (0.0) | 0/0 (0.0) | 1/1 (16.7) | 1/1 (2.0) |
| **Reproductive system and breast disorders** | 2/2 (25.0) | 0/0 (0.0) | 0/0 (0.0) | 0/0 (0.0) | 0/0 (0.0) | 0/0 (0.0) | 0/0 (0.0) | 0/0 (0.0) | 0/0 (0.0) | 2/2 (3.9) |
| Dysmenorrhea | 1/1 (12.5) | 0/0 (0.0) | 0/0 (0.0) | 0/0 (0.0) | 0/0 (0.0) | 0/0 (0.0) | 0/0 (0.0) | 0/0 (0.0) | 0/0 (0.0) | 1/1 (2.0) |
| Moderate | 1/1 (12.5) | 0/0 (0.0) | 0/0 (0.0) | 0/0 (0.0) | 0/0 (0.0) | 0/0 (0.0) | 0/0 (0.0) | 0/0 (0.0) | 0/0 (0.0) | 1/1 (2.0) |
| Nipple pain | 1/1 (12.5) | 0/0 (0.0) | 0/0 (0.0) | 0/0 (0.0) | 0/0 (0.0) | 0/0 (0.0) | 0/0 (0.0) | 0/0 (0.0) | 0/0 (0.0) | 1/1 (2.0) |
| **Cardiac disorders** | 0/0 (0.0) | 1/1 (50.0) | 0/0 (0.0) | 0/0 (0.0) | 0/0 (0.0) | 0/0 (0.0) | 0/0 (0.0) | 0/0 (0.0) | 0/0 (0.0) | 1/1 (2.0) |
| Palpitations | 0/0 (0.0) | 1/1 (50.0) | 0/0 (0.0) | 0/0 (0.0) | 0/0 (0.0) | 0/0 (0.0) | 0/0 (0.0) | 0/0 (0.0) | 0/0 (0.0) | 1/1 (2.0) |
| **Ear and labyrinth disorders** | 0/0 (0.0) | 0/0 (0.0) | 0/0 (0.0) | 0/0 (0.0) | 0/0 (0.0) | 0/0 (0.0) | 0/0 (0.0) | 0/0 (0.0) | 1/1 (16.7) | 1/1 (2.0) |
| Tinnitus | 0/0 (0.0) | 0/0 (0.0) | 0/0 (0.0) | 0/0 (0.0) | 0/0 (0.0) | 0/0 (0.0) | 0/0 (0.0) | 0/0 (0.0) | 1/1 (16.7) | 1/1 (2.0) |

^*^AEs were classified according to MedDRA Version 21.0

Cohorts: PBO 1 = Placebo BID on Day 1 and QD on Days 2 to 7 IV infusion; PBO 2 = Placebo BID on Days 1 and 2 and QD on Days 3 to 7 IV infusion; PBO 3 = Placebo BID on Day 1 and QD on Days 2 to 7 IV infusion with ondansetron; A = 1500 mg BID on Day 1 and 900 mg QD on Days 2 to 7 FMGX IV infusion; B = 900 mg BID on Days 1 and 2 and 900 mg QD on Days 3 to 7 FMGX IV infusion; C = 1000 mg BID on Day 1 and 900 mg QD on Days 2 to 7 FMGX IV infusion with ondansetron; D = 1000 mg BID on Day 1 and 750 mg QD on Days 2 to 7 FMGX IV infusion; E = 1000 mg BID on Day 1 and 850 mg QD on Days 2 to 7 FMGX IV infusion; F = 1000 mg BID on Day 1 and 900 mg QD on Days 2 to 7 FMGX IV infusion

% = percentage of the total number of participants per treatment that experienced the AEs; AE = adverse events; BID, twice daily; E = number of times the AEs occurred; FMGX = fosmanogepix; IV = intravenous; N = number of participants exposed; n = number of participants that experienced the AEs; PBO = placebo; PT = preferred term; QD, once daily; SOC = system organ class; TEAEs = treatment-emergent adverse events
